# Pd‐Catalyzed Asymmetric Dearomative Heck/Tsuji‐Trost Difunctionalization of Naphthalenes

**DOI:** 10.1002/advs.76459

**Published:** 2026-07-13

**Authors:** Long‐Ling Ma, Bing Xu, Junliang Zhang, Zhan‐Ming Zhang

**Affiliations:** ^1^ Department of Chemistry, State Key Laboratory of Green Chemical Synthesis and Conversion Fudan University Shanghai P. R. China; ^2^ School of Pharmacy Naval Medical University Shanghai P. R. China; ^3^ State Key Laboratory of Organometallic Chemistry Shanghai Institute of Organic Chemistry Chinese Academy of Sciences Shanghai P. R. China; ^4^ School of Chemistry and Chemical Engineering Henan Normal University Xinxiang Henan P. R. China; ^5^ Fudan Zhangjiang Institute Shanghai P. R. China

**Keywords:** asymmetric Heck/Tsuji‐Trost reaction, dearomatization of naphthalenes, palladium‐catalyzed

## Abstract

Dearomative reactions represent one of the crucial strategies for constructing molecular complexity, while the asymmetric 1,4‐difunctionalization of naphthalenes, which enables efficient construction of two distal chiral centers, requires further exploration. Herein, we report a Pd‐catalyzed asymmetric dearomative Heck/Tsuji‐Trost reaction, which achieves the 1,4‐difunctionalization of the non‐activated naphthalenes for the synthesis of a series of spirocyclic products bearing two distal chiral centers. Through the employment of the new chiral sulfinamide phosphine ligand featuring bulky steric hindrance, the reaction system exhibits excellent regio‐, enantio‐, and diastereoselectivity. 4‐Aminospirocyclohexene oxindoles with diverse functional groups were obtained in moderate to excellent yields with good to excellent enantioselectivity. The reaction proceeds under mild conditions with versatile transformations of the products. Moreover, a plausible reaction mechanism was proposed based on the mechanistic studies.

## Graphical abstract

We have developed a palladium‐catalyzed asymmetric dearomative Heck/Tsuji‐Trost 1,4‐difunctionalization reaction of naphthalenes, affording 4‐aminospirocyclohexene oxindole products bearing two remote chiral centers. Through the effect of the newly designed chiral sulfinamide phosphine ligand with large steric hindrance, side reactions are inhibited, and excellent regio‐, enantio‐, and diastereoselectivity are achieved. The reaction proceeds under mild conditions, with yields and *ee* values reaching up to 97%.

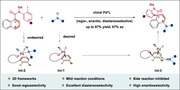

## 1 Introduction

Aromatic compounds, as abundant and fundamental chemical feedstocks, play a pivotal role in organic synthesis, pharmaceuticals, and industrial applications. Recently, the “escaping from the flatland” concept, which strategically transforms planar aromatic rings into three‐dimensional (3D), C(sp^3^)‐enriched frameworks, has become a crucial principle in modern medicinal chemistry that guides the modification of drug structures and the development of new drugs [1, 2, 3, 4, 5, 6]. Notably, dearomative reactions stand out as one of the important and efficient strategies for building molecular complexity, enabling the direct conversion of planar aromatic building blocks into 3D, functionalized compounds.

In previous studies, dearomatization reactions of indole [7, 8, 9, 10, 11, 12, 13], naphthol, and phenol [14, 15, 16, 17, 18, 19, 20, 21, 22, 23, 24, 25, 26] have been achieved. The research groups of You, Jia, and Luan have made outstanding contributions to this field. As the electrically unbiased aromatic structure, the naphthalenes and benzenes possess higher aromatic stabilization energy compared to heteroarenes [27], the dearomatization of the non‐activated arenes still remains a challenge. As disclosed recently by the groups of You, Jia, Yamaguchi, Zhou, Guo, and Luan, some cases of dearomative reactions of naphthalenes and benzenes [28, 29, 30, 31, 32, 33, 34, 35, 36, 37, 38, 39] have been achieved. Among them, some cases of dearomative 1,2/1,4‐addition of naphthalenes and benzenes have been reported. However, most of the present studies fail to achieve the asymmetric dearomative reaction. Our group previously achieved the Pd‐catalyzed enantioselective dearomatization Mizoroki−Heck reaction of naphthalenes, which constructed the spiro products with one single chiral center [34]. Inspired by this work, we herein report an asymmetric dearomative 1,4‐difunctionalization of naphthalenes, which enables the construction of two remote chiral centers in a single transformation. The reaction mechanism proceeds as follows: initial oxidative addition of the substrates containing a halogen, followed by dearomative migratory insertion, generates an alkyl‐Pd intermediate, then through the migration of Pd, generating the allylpalladium intermediate. The intermediate is captured by nucleophiles, ultimately yielding the product while regenerating the Pd catalyst. Combining dearomative reactions with the asymmetric Heck/Tsuji‐Trost 1,4‐difunctionalization of naphthalenes allows for the efficient and direct synthesis of valuable chiral 3D molecules starting from simple planar aromatic compounds.

**FIGURE 1:**
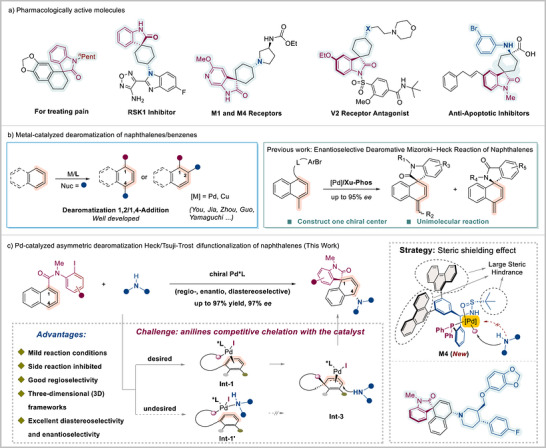
Background and discovery. (a) Selected pharmacologically active molecules possessing spirooxindole cores. (b) The metal‐catalyzed dearomatization of naphthalenes/benzenes. (c) This work: Pd‐catalyzed asymmetric dearomative Heck/Tsuji‐Trost difunctionalization of naphthalenes.

As a key subtype of spirooxindoles, 4‐aminospirocyclohexane(‐ene) oxindoles are widely distributed in bioactive natural alkaloids and pharmaceutically relevant compounds (Figure 1a) [40, 41, 42, 43]. Given their significance, we have conducted research on the asymmetric dearomatization Heck/Tsuji‐Trost difunctionalization of naphthalene with alkyl/aryl amines, aiming to synthesize structurally modified high‐value 4‐aminospirocyclohexene oxindole compounds. This reaction faces the following challenges: (i) the existence of multiple competing reactions, such as the Buchwald‐Hartwig amination reaction, C‐H arylation reaction, and Heck reaction; (ii) the need for precise control of the reaction's regio‐, enantio‐ and diastereoselectivity; (iii) anilines may undergo competitive chelation with the catalyst, thereby leading to a decrease in the reaction efficiency and selectivity (chemical, stereoselectivity, and regioselectivity). Notably, as mentioned earlier, the **Xu‐Phos** previously used by our group was incompatible with this reaction system. Herein, employing our own developed Sadphos as the chiral ligand [44, 45, 46, 47, 48, 49, 50, 51, 52] and using our newly designed chiral sulfinamide phosphine ligand featuring bulky steric hindrance, we effectively regulated the enantioselectivity of the reaction via a steric shielding effect strategy [53, 54, 55, 56]. This not only enabled the acquisition of an exclusive configuration product bearing two chiral centers but also significantly suppressed the formation of undesired byproducts (Figure 1c). Accordingly, a diverse array of chiral cyclohexenamines featuring a spirooxindole scaffold was synthesized, achieving moderate to excellent yields alongside good to excellent enantioselectivity.

## 2 Results and Discussion

To initiate our work, **1a** and methylbenzylamine **2a** were selected as model substrates to evaluate various ligands under the conditions of PdCl_2_ (5 mol%), Ag_3_PO_4_ (1.0 equiv), Na_2_HPO_4_⚫12H_2_O (2 equiv) in DMA at 120°C for 12 h (Table 1). Among the commercially available ligands, **L3** could deliver the target product in 33% yield with a low *ee* value. **L1** and **L2** could afford high yield (89% yield) with −23% *ee* and 40% *ee*. Subsequently, we screened a series of chiral sulfonamide phosphine (**Sadphos**) ligands, including **Ming‐Phos**, **Xu‐Phos**, **PC‐Phos**, **TY‐Phos**, **Xiang‐Phos**, **Xiao‐Phos**, and **Wei‐Phos**, all of which successfully generated the target product. However, when **PC‐Phos** was employed, only the racemic product was obtained in 15% yield. The use of **Xu‐Phos** afforded the desired product in 43% yield with 31% *ee*. In contrast, **TY‐Phos**, **Xiang‐Phos**, **Xiao‐Phos**, and **Wei‐Phos** only delivered the product with low *ee* values. Upon screening **Ming‐Phos**, the product **3a** was obtained in 67% yield with a relatively significantly improved *ee* value (45% *ee*). Notably, the further modification of the **Ming‐Phos** skeleton revealed that increasing the steric bulk of the 3,5‐substituents on the phenyl ring enhanced enantioselectivity. For instance, **M2** resulted in 52% yield and 74% *ee*, while the higher *ee* value (81% *ee*) was observed for sterically more hindered ligand **M3**. When the **M4** bearing 9‐phenanthryl group was used, the target product **3a** was obtained in 66% yield and 83% *ee*. Consequently, **M4** was identified as the optimal ligand for this transformation.

**TABLE 1:** Optimization from ligands, solvents, palladium, bases, and temperatures.

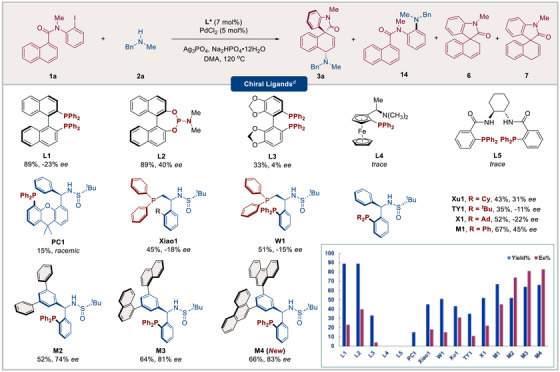						
Entry	Alteration to conditions	Yield of **3a** (%) ^b^	*Ee* (%) ^c^	Yield of **14** (%)^b^	Yield of **6** (%)^b^	Yield of **7** (%)^b^
1^a^	None	76	97	—	—	—
2	Pd(OAc)_2_	trace	—	—	4	4
3	Pd(TFA)_2_	32	31	8	2	7
4	Pd_2_(dba)_3_	60	76	2	—	3
5	Pd_2_(dba)_3_·CHCl_3_	59	86	3	—	5
6	*^t^* BuOK	6	62	2	4	6
7	NaHCO_3_	86	84	—	—	2
8	KF	74	91	2	6	8
9	CH_3_COOLi	67	14	3	—	7
10	DBU	trace	—	—	2	—
11	THF	86	91	—	—	—
12	Tol	31	80	4	3	3
13	CH_3_CN	88	74	—	—	2
14	EA	68	78	2	2	2
15	MBTE	44	90	3	3	2
16	120°C	87	88	—	4	4
17	90°C	81	93	—	3	2

^a^ Standard conditions: **1a** (0.2 mmol), **2a** (0.4 mmol), **M4** (7 mol%), [*η*‐PhC_3_H_4_PdCl]_2_ (2.5 mol%), Ag_3_PO_4_ (0.2 mmol), Na_2_HPO_4_•12H_2_O (0.4 mmol) in 1.0 mL DCM at 80°C for 12 h.

^b^ GC yield with 1,3‐dimethoxybenzene as an internal standard.

^c^ Enantioselectivity was determined by chiral HPLC.

^d^ **1a** (0.2 mmol), **2a** (0.4 mmol), L* (7 mol%), PdCl_2_ (5 mol%), Ag_3_PO_4_ (0.2 mmol), Na_2_HPO_4_•12H_2_O (0.4 mmol) in 1.0 mL DMA at 120°C for 12 h.

Afterward, **M4** was employed as the optimal ligand, and the influence of the Pd source, base, solvent, and reaction temperature were conducted. Identifying [*η*‐PhC_3_H_4_PdCl]_2_, Ag_3_PO_4_, Na_2_HPO_4_⚫12H_2_O, DCM, and 80°C as the optimal conditions that delivered 76% yield and 97% *ee*. The choice of Pd source significantly influenced the reaction. Pd(OAc)_2_ failed to deliver the target product. When Pd_2_(dba)_3_ and Pd_2_(dba)_3_⚫CHCl_3_ were employed as the palladium source, both the yield and enantioselectivity slightly decreased, affording the product in 59%−60% yield with 76%−86% *ee*. The use of Pd(TFA)_2_ led to a significant decline in both yield and enantioselectivity, providing **3a** in only 32% yield and 31% *ee* (Table 1, entries 2−5). Moreover, the effect of various bases were examined (Table 1, entries 6−10). In particular, DBU completely inhibited product formation (Table 1, entry 10), whereas **3a** could be afforded in high yields (74%−86%) with excellent enantioselectivity (84%−91% *ee*) through the use of NaHCO_3_ or KF (Table 1, entries 7−8). The stronger base *^t^* BuOK delivered moderate enantioselectivity but severely compromised the yield (Table 1, entry 6). CH_3_COOLi enabled product formation with 67% yield, but the *ee* value was markedly low (13% *ee*). We then conducted the effect of solvent. Solvent evaluation revealed that THF and MTBE could lead to high *ee* values (90%−91% *ee*) (Table 1, entries 11, 15). Other solvents (toluene, CH_3_CN, EA) were tested; the corresponding product was delivered in 31%−88% yield with 74%−80% *ee*. Furthermore, we investigated the influence of reaction temperature. At 120°C, the reaction afforded 87% yield and 88% *ee* (Table 1, entry 15). Reducing the temperature from 90°C to 80°C enhanced the enantioselectivity from 93% to 97% *ee* (Table 1, entries 1, 17). As the temperature decreased, a slight reduction in yield was observed, accompanied by an increase in the *ee* value. Finally, 80°C was selected as the optimal reaction temperature.

**FIGURE 2:**
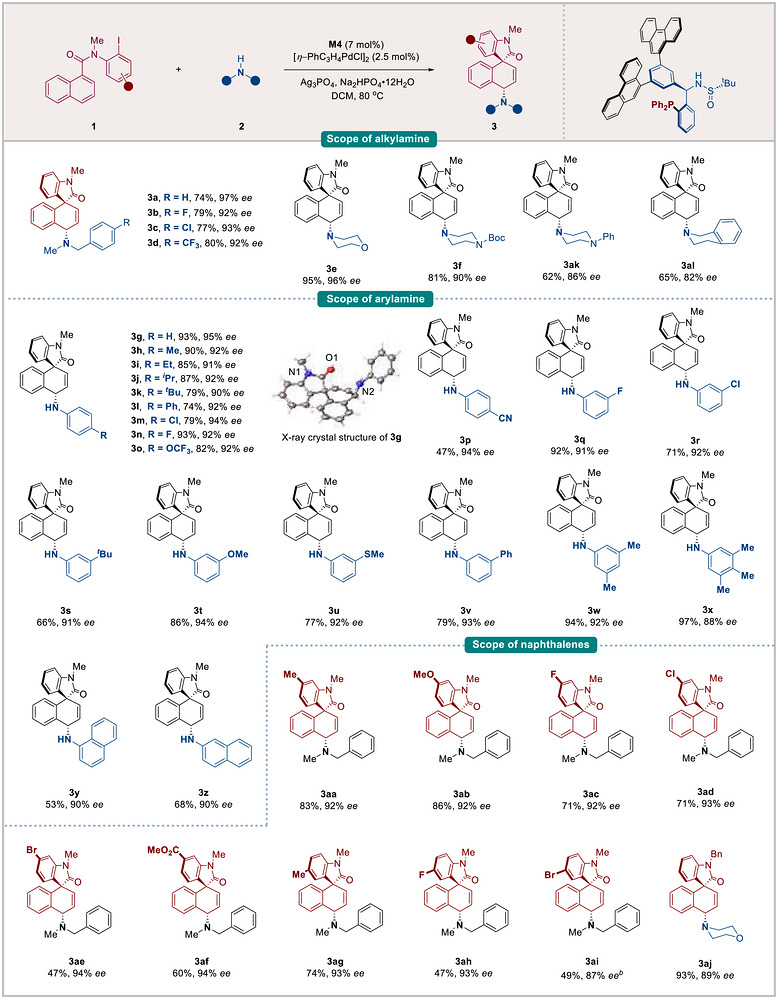
Scope of the Substrates. (a) Unless otherwise noted, all reactions were performed with **1** (0.3 mmol), **2/4** (0.6 mmol), Ag_3_PO_4_ (0.3 mmol), Na_2_HPO_4_•12H_2_O (0.6 mmol), [*η*‐PhC_3_H_4_PdCl]_2_ (2.5 mol%) and **M4** (7 mol%) in 1.5 mL DCM at 80°C for 15 h; (b) 100°C.

Under the optimized conditions, substrate scope investigations were conducted (Figure 2). For amine substrates, both alkylamines and arylamines were explored. Various substituents at the *para* position of methylbenzylamine, including halide (F, Cl) and the electron‐withdrawing group CF_3_, were well tolerated, affording products **3b**−**3d** in 77%−80% yield and 92%−93% *ee*. Cyclic secondary alkylamines such as morpholine and N‐Boc‐piperazine were viable reaction partners, delivering **3e** and **3f** in 81%−95% yield and 90%−96% *ee*. In addition, when 1‐phenylpiperazine and 1,2,3,4‐tetrahydroisoquinoline were employed as substrates, **3ak** was obtained in 62% yield with 86% ee, and **3al** was afforded in 65% yield with 82% ee.

Furthermore, the applicability of variously substituted arylamines was examined. Aniline as the substrate afforded product **3** **g** in excellent yield and enantioselectivity (93% yield, 95% *ee*). The absolute configuration of **3** **g** was confirmed by single‐crystal x‐ray diffraction and could be extrapolated to other products. For *para*‐substituted arylamines, the substrates bearing the electron‐donating groups such as alkyl (Me, Et, *^i^* Pr, *^t^* Bu) and trifluoromethoxy led to **3h**−**3k**, **3o** in 79%−90% yield and 90%−92% *ee*. On the other hand, electron‐withdrawing groups including phenyl and cyano (**3l**, **3p**, **3v**) were compatible, providing excellent *ee* (92%−94% *ee*), though **3p** exhibited moderate yield. The diminished yield of **3p** may correlate with the reduced nucleophilicity of the arylamine, consistent with the electron‐withdrawing character of the cyano group. Halide (F, Cl) at the *para* and *meta* positions afforded robust performance; the corresponding products (**3m**−**3n**, **3q**−**3r**) were formed in 71%−93% yield and 91%−94% *ee*. For the *meta*‐substituted substrates, the tert‐butyl group with steric hindrance afforded the corresponding product **3s** with high yield (66% yield) and excellent enantioselectivity (91% *ee*). Remarkably, the electron‐withdrawing substituents such as methoxy and methylthio groups (**3t**−**3u**) demonstrated excellent performance, yielding products in 77%−86% yield with 92%−94% *ee*. We also examined poly‐substituted arylamines. **3w** was obtained in high yield (94% yield) with high ee value (92% *ee*). The trisubstituted aryl amines (**3x**) were viable reaction partners, delivering outstanding yield (97% yield) despite a modest decrease in enantioselectivity (88% *ee*). Moreover, moderate yields (53%−68%) and good ee values (90% *ee*) were observed when naphthylamines were treated in the reaction (**3y**−**3z**). Notably, *p*‐nitroaniline and heterocyclic amines containing furan, thiophene, and quinoline moieties failed to deliver the target products. This result may be ascribed to the fact that the nitro group greatly decreases the nucleophilicity of aniline, and heterocycles exert a poisoning effect on palladium catalysts. (see Supporting Information for details).

**FIGURE 3:**
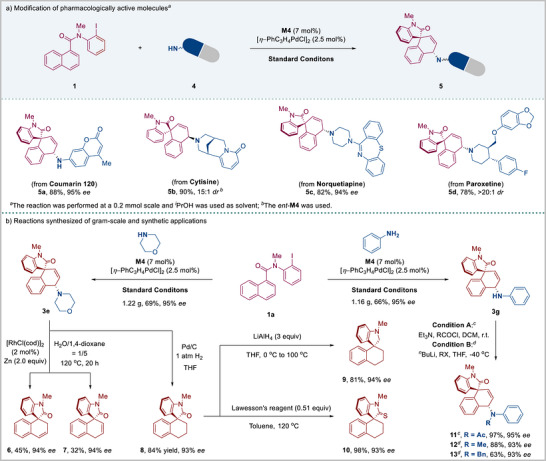
The synthetic transformation. a, modification of pharmacologically active molecules. b, gram‐scale and functionalization of **3e** and **3** **g**.

For naphthalene derivatives **1**, we investigated the substituents at the 3‐ and 4‐positions of the phenyl ring. The substrates exhibited tolerance toward electron‐donating groups (Me, OMe), affording products **3aa**−**3ab** and **3ag** in 74%−86% yield with 92%−93% *ee*. In contrast, the substrates with an electron‐withdrawing group (COOMe) participated smoothly in the reaction, and the corresponding product **3af** was obtained in moderate yield with excellent enantioselectivity (60% yield, 94% *ee*). The halide substituents (F, Cl, Br) on the phenyl ring could be capable of the reaction effectively, delivering the corresponding products **3ac**−**3ae** and **3ah** with 92%−94% *ee*. However, substrates with *N*‐*para* substitution generally exhibited lower reactivity compared to the *N*‐*ortho* substituted counterparts. For instance, the substrate bearing a Br substituent at the *para* position to the N atom (**3ai**) required heating to 100°C, resulting in only a moderate yield. Owing to the elevated temperature, the *ee* value slightly decreased, affording product **3ai** with 87% *ee*. When the methyl group on the nitrogen of substrate **1** was replaced by a benzyl group, the substrate was also smoothly converted into the target product **3aj** in 93% yield with 89% ee. It is noteworthy that the approach demonstrated excellent compatibility with amines derived from natural products and pharmaceutical molecules, including coumarin 120, cytisine, norquetiapine, and paroxetine, affording the corresponding products in 78%−90% yields (Figure 3a). For substrates containing chiral centers, **5b** and **5d** were obtained with 15:1 *dr* and > 20:1 *dr*, while high *ee* values were achieved for **5a** and **5c**.

To evaluate the practicality of this strategy, we conducted gram‐scale dearomative Heck/Tsuji‐Trost reaction of **2e** (5 mmol) and **2** **g** (5 mmol) under the standard conditions, delivering the corresponding products **3e** in 69% yield with 95% *ee* and **3** **g** in 66% yield with 95% *ee* respectively (Figure 3b). The dearomatized products were subsequently subjected to some synthetic transformations. Two types of reduction reactions can be performed on **3e**. Catalytic reduction with [RhCl(cod)]_2_ afforded products **6** and **7** bearing a double bond in 45% and 32% yield, respectively, with both 94% *ee* [57]. In an atmosphere of H_2_, Pd/C catalyzed hydrogenation of **3e** delivered **8** in 84% yield with 93% *ee*, which can undergo further transformations. Reduction of the amide group with LiAlH_4_ yielded **9** in 81% yield with 94% *ee*. Treatment with Lawesson's reagent afforded product **10** in 98% yield with 93% *ee*. For **3** **g**, the acetylation of the N─H bond was achieved under mild conditions to furnish product **11** in 97% yield without erosion of the *ee* value. Methylation of **3** **g** of delivered product **12** in 88% yield with 93% *ee* and reaction of **3** **g** with benzyl bromide provided product **13** in 63% yield with 93% *ee*.

**FIGURE 4:**
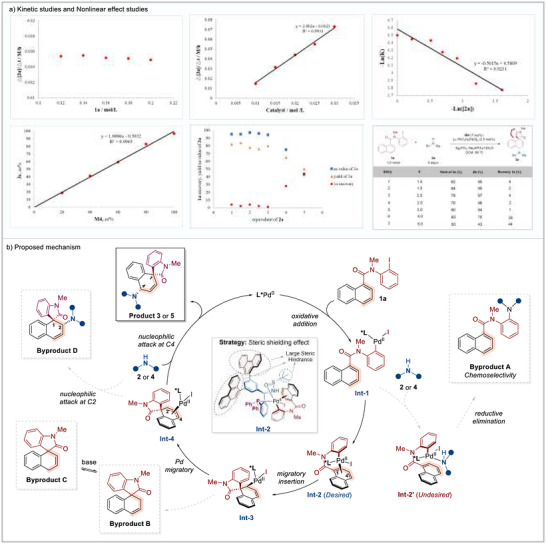
Kinetic Studies and Proposed Mechanism.

To gain deeper mechanistic insights, we performed a series of experiments (Figure 4a). Investigation of the nonlinear effect between the ligand and product revealed a linear correlation between the enantiomeric composition of chiral ligand **M4** and the *ee* of product **3a**, suggesting a 1:1 Pd/**M4** coordination in the catalytic cycle. Subsequently, kinetic studies were conducted under optimized conditions using **1a** and **2a** as substrates. The results demonstrated the first‐order dependence of the concentration of the palladium catalyst, the zero‐order dependence on the concentration of naphthalene derivative **1a**, and the negative fractional‐order dependence on the concentration of methylbenzylamine **2a**. Besides, a significant amount of substrate **1** was observed when the amine concentration was high. Based on the experimental results, we have made the following speculations. At an elevated concentration of **2a**, intermediate **int‐2'** is generated; **2a** would coordinate with Pd, which hinders the migratory insertion process, thereby reducing the overall reaction rate [58].

A plausible reaction mechanism was proposed [59, 60] (Figure 4b). As shown, initially, the naphthalene derivative **1a** would undergo oxidative addition to generate the aryl palladium intermediate **Int‐1**. Subsequently, coordination of Pd with naphthalene affords intermediate **Int‐2**. Intramolecular migratory insertion then leads to the formation of the palladium intermediate **Int‐3**. Then **Int‐3** would undergo palladium migration, resulting in the formation of allylpalladium intermediate **Int‐4**. Nucleophilic attack of the amine at the C4 of **Int‐4** affords the target product with concomitant regeneration of the palladium catalyst. The key steps for the catalytic system to control the two chiral centers of the product are as follows: the chiral configuration at C1 is determined by the formation of **Int‐2** and the migratory insertion process, while the chiral configuration at C4 is governed by the nucleophilic attack of the amine on **Int‐4**. The cooperative effect of the two phenanthrene moieties and the *tert*‐butyl group in the structure of the ligand **M4** generates large steric hindrance, which is crucial for chiral control. Additionally, the reaction mechanism reveals other plausible reaction pathways. Coordination of the amine with Pd in **Int‐1,** followed by reductive elimination, gives byproduct **A**. The intermediate **Int‐3** undergoes protonation to yield the reductive Heck byproduct **B**, which can interconvert with byproduct **C** under the influence of a base. Nucleophilic attack of the amine at the C2 position of **Int‐4** gives byproduct **D**.

## 3 Conclusions

In summary, we have achieved a Pd‐catalyzed asymmetric dearomative Heck/Tsuji‐Trost 1,4‐difunctionalization reaction of naphthalenes. Notably, both alkyl and aryl amines serve as viable reaction partners. The new chiral ligand **M4** played a pivotal role in controlling the regio‐, enantio‐, and diastereoselectivity of the reaction. Through this strategy, various 4‐aminospirocyclohexene oxindoles were obtained with up to 97% *ee*. Furthermore, a plausible reaction mechanism has been proposed based on experimental observations, offering insights into the reaction pathway. This strategy integrates dearomative reactions with the 1,4‐difunctionalization of naphthalenes, providing an efficient approach for the synthesis of 4‐aminospirocyclohexane(‐ene) oxindole scaffolds.

## Author Contributions

**Long‐Ling Ma**: writing – original draft, methodology, data curation. **Junliang Zhang**: resources, funding acquisition, writing – review and editing, supervision, project administration. **Zhan‐Ming Zhang**: conceptualization, supervision, writing – review and editing, funding acquisition, project administration. **Bing Xu**: data curation, writing – original draft, writing – review and editing.

## Conflicts of Interest

The authors declare no conflicts of interest.

## Supporting information




**Supporting File**: advs76459‐sup‐0001‐SuppMat.docx.

## Data Availability

The data that supports the findings of this study are available in the supplementary material of this article.
