# Supplementary material for: Pd‐Catalyzed Asymmetric Dearomative Heck/Tsuji‐Trost Difunctionalization of Naphthalenes
Source: Adv Sci (Weinh). 2026 Jul 13;13(54):e76459. doi: 10.1002/advs.76459 (PMC13360129; doi:10.1002/advs.76459)
Supplement: Supplementary file 1 [file ADVS-13-e76459-s001.docx]

*Supporting Information*

**Palladium-catalyzed asymmetric dearomative Heck/anion-capture reaction of naphthalenes and amines**

Long-Ling Ma,^1^ Bing Xu,^2^ Junliang Zhang,^1,4,5,6^* and Zhan-Ming Zhang^1,3,5^*

^1^Department of Chemistry, Fudan University, 2005 Songhu Road, Shanghai, 200438, P. R. China.

^2^School of Pharmacy, Naval Medical University, Shanghai 200433, P. R. China.

^3^Fudan Zhangjiang Institute, Shanghai, 201203, P. R. China.

^4^State Key Laboratory of Organometallic Chemistry, Shanghai Institute of Organic Chemistry, Chinese Academy of Sciences, Shanghai, 200032, P. R. China.

^5^State Key Laboratory of Green Chemical Synthesis and Conversion.

^6^School of Chemistry and Chemical Engineering, Henan Normal University, Xinxiang, Henan, 453007, P. R. China.

**E-mail*: junliangzhang@fudan.edu.cn; zhanmingzhang@fudan.edu.cn

**Contents**

General Information	S1

Synthesis of new **Ming-Phos**	S2

Optimization of reaction conditions	S3

General experimental procedure and characterization of products	S8

Gram-scale synthesis	S52

Synthesis applications	S54

Non-linear effect experiments	S62

Kinetic Studies	S63

X-Ray Structure and Crystal Data of **3g**	S67

References	S74

^1^H , ^19^F, ^31^P, ^13^C NMR	S75

1. **General Information**

All reactions were carried out under an atmosphere of argon in sealed tube with magnetic stirring. ^1^H NMR spectra, ^19^F NMR spectra, ^31^P NMR spectra, ^13^C NMR spectra were recorded on a Bruker 400 MHz spectrometer in CDCl_3_. All signals are reported in ppm with the internal TMS signal at 0 ppm as a standard. Data for ^1^H NMR spectra are reported as follows: chemical shift (ppm, referenced to TMS; s = singlet, d = doublet, t = triplet, dd = doublet of doublets, m = multiplet), coupling constant (Hz), and intergration. Data for ^13^C NMR are reported in terms of chemical shift (ppm) relative to residual solvent peak (CDCl_3_: 77.0 ppm). Reactions were monitored by thin layer chromatography (TLC) using silica gel plates. Flash column chromatography was performed over silica gel (300-400 mesh). The substrates **1a, 1aa-1ai** were synthesized according to published procedures.^1^ The spectral data of the substrates were consisted with that reported in the literature. The solvents, substituted aniline and alkyl amines were used directly as received from commercial sources (Energy Chemical Co., Ltd., Adamas beta®, Bidepharm Co., Ltd.) without further purification. A Sadphos kit was purchased from Anhui Cat-Lab Technology Co., Ltd. The enantionmeric excesses of the products were determined by chiral stationary phase HPLC using a Chiralpak ODH, ADH, IA. Specific rotations were measured by PolAAr 3005 High Accuracy Polarimeter. Melting Point data were recorded on WRS-2.

2. **Synthesis of new Ming-Phos**

Under nitrogen atmosphere, to an oven-dried 250 mL three-necked flask equipped with a magnetic stir bar was charged with aryl bromide (1.5 equiv) and dry THF. The mixture was cooled to -78 ^o^C and *^n^*BuLi (1.5 equiv, 2.5 M in hexane) was added dropwise. After stirred at -78 ^o^C for 1 h, the chiral sulfinyl imine (1.0 equiv), which was prepared according to the literature procedure^2^, in dry THF was added. The reaction mixture was kept at -78 ^o^C for 0.5 h and then slowly warmed to room temperature. Upon reaction completion, the reaction was quenched by saturated NH_4_Cl solution and extracted with ethyl acetate for 3 times. The combined organic layers were washed by brine, dried over Na_2_SO_4_ and concentrated to dryness. The residue was purified by column chromatography (petroleum ether/ethyl acetate = 5/1) to afford **Ming-Phos**.

Synthesis of (*R*)-*N*-((*R*)-(3,5-di(phenanthren-9-yl)phenyl) (2-(diphenylphosphaneyl)phenyl)methyl)-2-methylpropane-2-sulfinamide (**M4**)

White solid. 2.46 g, 63.9% yield. ^1^H NMR (400 MHz, CDCl_3_) δ 8.76 (d, *J* = 8.3 Hz, 2 H), 8.71 (d, *J* = 8.1 Hz, 2 H), 7.94 (d, *J* = 8.2 Hz, 2 H), 7.89 (d, *J* = 7.6 Hz, 2 H), 7.73 (dd, *J* = 7.9, 4.4 Hz, 1 H), 7.65 (q, *J* = 6.0, 4.5 Hz, 5 H), 7.60 (d, *J* = 6.3 Hz, 3 H), 7.52 (d, *J* = 7.7 Hz, 5 H), 7.39 (t, *J* = 7.6 Hz, 1 H), 7.34 – 7.24 (m, 4 H), 7.20 (t, *J* = 7.4 Hz, 2 H), 7.12 – 7.00 (m, 3 H), 6.96 (t, *J* = 7.5 Hz, 2 H), 6.87 (t, *J* = 7.4 Hz, 1 H), 6.79 (dd, *J* = 8.1, 4.0 Hz, 1 H), 4.14 (s, 1 H), 1.24 (s, 9 H). ^13^C NMR (101 MHz, CDCl_3_) δ 147.46 (d, *J* = 24.1 Hz), 141.23, 140.67, 138.21, 135.96 (d, *J* = 7.1), 135.48 (d, *J* = 16.3), 135.23, 133.87, 133.69 (d, *J* = 3.6 Hz), 133.52, 131.48, 130.88, 130.63, 130.49, 129.94, 129.83, 129.14, 128.62, 128.53, 128.46, 128.39, 128.28, 128.21, 127.96, 127.88 (d, *J* = 4.2), 127.69, 126.96, 126.83, 126.60, 126.50, 126.43, 122.87, 122.50, 59.71 (d, *J* = 26.2 Hz), 56.17, 22.79. ^31^P NMR (162 MHz, CDCl_3_) δ -18.83. [α]_D_^20^ = 45.7 (*c* = 0.3, CHCl_3_). HRMS (ESI) calculated for C_57_H_47_NOPS: 824.3110 (M+H^+^), found: 824.3123.

3. **Optimization of reaction conditions**

**3.1 Optimization of ligands*^a^*.**

**3.2 Optimization of palladium salts*^a^*.**

| Entry | [Pd] | **3a** | | | Yield of **14**/%^[b]^ | Yield of **6**/%^[b]^ | Yield of **7**/%^[b]^ |
| --- | --- | --- | --- | --- | --- | --- | --- |
|  |  | Yield/% ^[b]^ | *Ee*/% ^[c]^ | |  |  |  |
| 1 | Pd(OAc)_2_ | trace | | - | - | 4 | 4 |
| 2 | Pd(TFA)_2_ | 32 | | 3- | 8 | 2 | 7 |
| 3 | Pd(dba)_2_ | 44 | | 78 | 4 | - | 3 |
| 4 | Pd_2_(dba)_3_ | 60 | | 76 | 2 | - | 3 |
| 5 | Pd_2_(dba)_3_·CHCl_3_ | 59 | | 86 | 3 | - | 5 |
| 6 | PdCl_2_ | 28 | | 35 | 2 | - | 2 |
| 7 | [Pd(allyl)Cl]_2_ | 63 | | 70 | 2 | - | 3 |
| 8 | Bis(2,2,6,6-Tetramethylheptanedionato)palladium(II)   | trace | | - | - | 2 | 3 |
| 9 | Dichloro(norbornadiene)  palladium(II)   | 42 | | 75 | 2 | - | 2 |
| 10 | Pd(COD)Cl_2_ | 42 | | 66 | 2 | - | 2 |
| 11 | [*η*-PhC_3_H_4_PdCl]_2_ | 76 | | 97 | - | - | - |

***^a^*Unless otherwise noted, all reactions were performed with 0.2 mmol of 1a, 0.4 mmol of 2a, 0.2 mmol of Ag_3_PO_4_, 0.4 mmol of** Na_2_HPO_4_·12H_2_O, 5 mol% [Pd] and 7 mol% of **M4** in 1.0 ml DCM at 80 ^o^C for 12 h; *^b^*GC yield with 1,3-dimethoxybenzene as an internal standard; *^c^*Enantioselectivity Determined by chiral-phase HPLC.

**3.3 Optimization of bases*^a^*.**

| Entry | Base | **3a** | | Yield of **14**/%^[b]^ | Yield of **6**/%^[b]^ | Yield of **7**/%^[b]^ |
| --- | --- | --- | --- | --- | --- | --- |
|  |  | Yield/% ^[b]^ | *Ee*/% ^[c]^ |  |  |  |
| 1 | NaHCO_3_ | 86 | 84 | - | - | 2 |
| 2 | *^t^*BuOK | 6 | 62 | 2 | 4 | 6 |
| 3 | *^t^*BuONa | 13 | 63 | - | 4 | 5 |
| 4 | *^t^*BuOLi | trace | - | - | 4 | 6 |
| 5 | CH_3_COOLi | 67 | 14 | 3 | - | 7 |
| 6 | KF | 74 | 91 | 2 | 6 | 8 |
| 7 | Na_2_CO_3_ | 94 | 74 | 2 | 3 | 2 |
| 8 | Li_2_CO_3_ | 80 | 86 | - | 6 | 3 |
| 9 | K_2_CO_3_ | trace | - | - | - | 3 |
| 10 | DBU | trace | - | - | 2 | - |
| 11 | Na_2_HPO_4_·12H_2_O | 76 | 97 | - | - | - |

***^a^*Unless otherwise noted, all reactions were performed with 0.2 mmol of 1a, 0.4 mmol of 2a, 0.2 mmol of Ag_3_PO_4_, 0.4 mmol of** base, 2.5 mol% [*η*-PhC_3_H_4_PdCl]_2_ and 7 mol% of **M4** in 1.0 ml DMA at 120 ^o^C for 12 h; *^b^*GC yield with 1,3-dimethoxybenzene as an internal standard; *^c^*Enantioselectivity Determined by chiral-phase HPLC.

**3.4 Optimization of solvent*^a^*.**

| Entry | Solvent | **3a** | | Yield of **14**/%^[b]^ | Yield of **6**/%^[b]^ | Yield of **7**/%^[b]^ |
| --- | --- | --- | --- | --- | --- | --- |
|  |  | Yield/% ^[b]^ | *Ee*/% ^[c]^ |  |  |  |
| 1 | THF | 86 | 91 | - | - | - |
| 2 | Tol | 31 | 80 | 4 | 3 | 3 |
| 3 | CH_3_CN | 88 | 74 | - | - | 2 |
| 4 | EA | 68 | 78 | 2 | 2 | 2 |
| 5 | MTBE | 44 | 90 | 3 | 3 | 2 |
| 6 | *^i^*PrOH | 84 | 89 | - | - | 2 |
| 7 | DMF | 88 | 81 | - | - | - |
| 8 | Et_2_O | 94 | 90 | 3 | 2 | 2 |
| 9 | dioxane | 82 | 91 | 2 | 2 | 2 |
| 10 | DMA | 86 | 89 | - | - | - |

***^a^*Unless otherwise noted, all reactions were performed with 0.2 mmol of 1a, 0.4 mmol of 2a, 0.2 mmol of Ag_3_PO_4_, 0.4 mmol of** Na_2_HPO_4_·12H_2_O, 2.5 mol% [*η*-PhC_3_H_4_PdCl]_2_ and 7 mol% of **M4** in 1.0 ml Solvent at 80 ^o^C for 12 h; *^b^*GC yield with 1,3-dimethoxybenzene as an internal standard; *^c^*Enantioselectivity Determined by chiral-phase HPLC.

**3.4 Optimization of** **equivalent of** **2a*^a^*.**

| Entry | X | **3a** | | Yield of **14**/%^[b]^ | Yield of **6**/%^[b]^ | Yield of **7**/%^[^^b]^ | **1a**/%^[b]^ |
| --- | --- | --- | --- | --- | --- | --- | --- |
|  |  | Yield/% ^[b]^ | *Ee*/% ^[c]^ |  |  |  |  |
| 1 | 1.0 | 82 | 95 | - | - | - | 4 |
| 2 | 1.5 | 84 | 95 | - | - | 2 | 2 |
| 3 | 2.0 | 78 | 97 | - | - | - | 4 |
| 4 | 2.5 | 76 | 96 | - | - | 3 | 2 |
| 5 | 3.0 | 80 | 94 | - | 2 | 4 | - |
| 6 | 4.0 | 65 | 75 | - | - | 4 | 28 |
| 7 | 5.0 | 50 | 43 | - | - | 2 | 44 |

***^a^*Unless otherwise noted, all reactions were performed with 0.2 mmol of** **1a, 0.2-1.0 mmol of** **2a, 0.2 mmol of Ag_3_PO_4_, 0.4 mmol of** Na_2_HPO_4_·12H_2_O, 2.5 mol% [*η*-PhC_3_H_4_PdCl]_2_ and 7 mol% of **M4** in 1.0 ml Solvent at 80 ^o^C for 12 h; *^b^*GC yield with 1,3-dimethoxybenzene as an internal standard; *^c^*Enantioselectivity Determined by chiral-phase HPLC.

1a recovery, yield/ee value of 3a

| equivalent of 2a | ee value of 3a | yield of 3a | 1a recovery |
| --- | --- | --- | --- |
| 1 | 95 | 82 | 4 |
| 1.5 | 95 | 84 | 2 |
| 2 | 97 | 78 | 4 |
| 2.5 | 96 | 76 | 2 |
| 3 | 94 | 80 | 1 |
| 4 | 75 | 65 | 28 |
| 5 | 43 | 50 | 44 |

**3.6 Optimization of temperature*^a^*.**

| Entry | T/^o^C | **3a** | | Yield of **14**/%^[b]^ | Yield of **6**%^[b]^ | Yield of **7**%^[b]^ |
| --- | --- | --- | --- | --- | --- | --- |
|  |  | Yield/% ^[b]^ | *Ee*/% ^[c]^ |  |  |  |
| 1 | 120 | 87 | 88 | - | 4 | 4 |
| 2 | 110 | 78 | 91 | - | 5 | 2 |
| 3 | 100 | 83 | 92 | - | 4 | 2 |
| 4 | 90 | 81 | 93 | - | 3 | 2 |
| 5 | 80 | 76 | 97 | - | - | - |

***^a^*Unless otherwise noted, all reactions were performed with 0.2 mmol of 1a, 0.4 mmol of 2a, 0.2 mmol of Ag_3_PO_4_, 0.4 mmol of** Na_2_HPO_4_·12H_2_O, 2.5 mol% [*η*-PhC_3_H_4_PdCl]_2_ and 7 mol% of **M4** in 1.0 ml DCM for 12 h; *^b^*GC yield with 1,3-dimethoxybenzene as an internal standard; *^c^*Enantioselectivity Determined by chiral-phase HPLC.

4. **General experimental procedure and characterization of products**

**Typical procedure for Pd-catalyzed enantioselective dearomative 1,4-difunctionalization of naphthalenes.**

Under nitrogen atmosphere, to an oven-dried 10 mL Schlenk tube equipped with a magnetic stir bar was added [*η*-PhC_3_H_4_PdCl]_2_ (3.8 mg, 0.0075 mmol, 2.5 mol%), **M4** (17.3 mg, 0.021 mmol, 7 mol%) and DCM (0.75 mL). The solution was stirred for 40 min at room temperature. Then **1** (0.3 mmol, 1.0 equiv), **2** or **4** (0.6 mmol, 2.0 equiv), Ag_3_PO_4_ (125.6 mg, 0.3 mmol, 1.0 equiv), Na_2_HPO_4_·12H_2_O (214.9 mg, 0.6 mmol, 2.0 equiv) and DCM (0.75 ml) were added to the solution. For **3a-3ah** and **4a-4d**, the mixture was then stirred at 80 ^o^C for about 15 h. For **3ai**, the mixture was stirred at 100 ^o^C for about 15 h. After the reaction was completed (monitored by TLC), the reaction mixture was concentrated to dryness and the residue was purified by column chromatography to afford desired product **3** or **5**.

The partially failed substrates of this reaction are shown in the figure.

4.1 Synthesis of (3*R*,4'*S*)-4'-(benzyl(methyl)amino)-1-methyl-4'*H*-spiro[indoline-3,1'-naphthalen]-2-one (**3a**)

Prepared according to typical procedure from **2a** (72.7 mg, 0.6 mmol), after a flash column chromatography (petroleum ether/ethyl acetate = 8/1) afforded the product **3a** as a pale yellow solid (84.2 mg, 74% yield) with 97% *ee*. Mp: 109-112 ^o^C. ^1^H NMR (400 MHz, CDCl_3_) δ 7.83 – 7.75 (m, 1H), 7.46 – 7.38 (m, 2H), 7.35 – 7.20 (m, 5H), 7.05 (td, *J* = 7.5, 1.4 Hz, 1H), 6.98 (td, *J* = 7.5, 1.0 Hz, 1H), 6.92 (d, *J* = 7.8 Hz, 1H), 6.86 (dd, *J* = 7.4, 1.3 Hz, 1H), 6.53 (dd, *J* = 7.9, 1.3 Hz, 1H), 6.39 (dd, *J* = 10.2, 3.3 Hz, 1H), 5.75 (dd, *J* = 10.2, 1.7 Hz, 1H), 4.57 (dd, *J* = 3.4, 1.7 Hz, 1H), 3.90 (d, *J* = 13.5 Hz, 1H), 3.66 (d, *J* = 13.5 Hz, 1H), 3.29 (s, 3H), 2.36 (s, 3H). ^13^C NMR (101 MHz, CDCl_3_) δ 177.66, 143.50, 140.27, 136.48, 135.65, 135.06, 129.25, 128.77, 128.39, 128.15, 127.82, 127.48, 127.40, 126.74, 126.45, 125.14, 124.55, 123.22, 108.10, 57.96, 57.88, 54.34, 38.09, 26.63. HRMS (EI) calculated for [C_26_H_24_N_2_O]^+^: 380.1889 found: 380.1883. Enantiomeric excess was determined by HPLC with a Chiralpak AD-H column (hexanes/2-propanol = 90/10, 1.0 mL/min, 254 nm); minor enantiomer *t_R_* = 7.7 min, major enantiomer *t_R_* = 13.8 min. [α]_D_^20^ = 107.2 (*c* = 0.3, CHCl_3_).

4.1.2 *N*-(2-(benzyl(methyl)amino)phenyl)-*N*-methyl-1-naphthamide (**14**)

Purified by the flash column chromatography (petroleum ether/ethyl acetate = 8/1), white solid. Mp: 46-48 ^o^C. ^1^H NMR (400 MHz, CDCl_3_) δ 8.30 (d, *J* = 8.5 Hz, 0.63H), 7.86 (t, *J* = 7.1 Hz, 0.72H), 7.72 (d, *J* = 8.1 Hz, 0.63H), 7.61 (d, *J* = 8.0 Hz, 0.65H), 7.47 (t, *J* = 7.9 Hz, 1.56H), 7.40 (dd, *J* = 15.7, 7.9 Hz, 1.8H), 7.35 – 7.22 (m, 4.11H), 7.18 (d, *J* = 7.6 Hz, 1.05H), 7.13 – 6.99 (m, 2.67H), 6.94 (t, *J* = 7.6 Hz, 0.7H), 6.83 (t, *J* = 7.5 Hz, 0.65H), 6.52 (d, *J* = 8.1 Hz, 0.62H), 4.30 (d, *J* = 53.8 Hz, 0.77H), 3.96 (s, 1.27H), 3.69 (s, 1.9H), 3.13 (s, 1.11H), 2.78 (s, 1.11H), 1.82 (s, 1.84H). ^13^C NMR (101 MHz, CDCl_3_) δ 170.85, 147.75, 137.48, 137.23, 133.61, 133.32, 131.08, 129.91, 129.59, 129.05, 128.90, 128.75, 128.39, 128.22, 128.05, 128.00, 127.71, 127.18, 127.05, 126.97, 126.24, 125.56, 125.31, 125.13, 124.84, 124.00, 123.46, 122.54, 121.56, 60.76, 59.92, 39.47, 38.04, 36.74. HRMS (ESI) calculated for C_26_H_25_N_2_O: 381.1961 (M+H^+^), found: 381.1965.

4.2 Synthesis of (3*R*,4'*S*)-4'-((4-fluorobenzyl)(methyl)amino)-1-methyl-4'*H*-spiro[indoline-3,1'-naphthalen]-2-one (**3b**)

Prepared according to typical procedure from **2b** (83.5 mg, 0.6 mmol), after a flash column chromatography (petroleum ether/ethyl acetate = 8/1) afforded the product **3b** as a yellow solid (93.9 mg, 79% yield) with 92% *ee*. Mp: 74-76 ^o^C. ^1^H NMR (400 MHz, CDCl_3_) δ 7.76 (d, *J* = 7.9 Hz, 1H), 7.36 – 7.23 (m, 4H), 7.05 (td, *J* = 7.5, 1.4 Hz, 1H), 7.02 – 6.94 (m, 3H), 6.92 (d, *J* = 7.8 Hz, 1H), 6.87 (dd, *J* = 7.4, 1.3 Hz, 1H), 6.53 (dd, *J* = 7.9, 1.3 Hz, 1H), 6.38 (dd, *J* = 10.2, 3.4 Hz, 1H), 5.75 (dd, *J* = 10.2, 1.6 Hz, 1H), 4.57 (s, 1H), 3.85 (d, *J* = 13.5 Hz, 1H), 3.58 (d, *J* = 13.5 Hz, 1H), 3.28 (s, 3H), 2.36 (s, 3H). ^13^C NMR (101 MHz, CDCl_3_) δ 177.58, 161.80 (d, *J* = 244.1 Hz), 143.48, 136.31, 135.98 (d, *J* = 3.1 Hz), 135.55, 135.08, 127.48, 127.46, 129.19, 128.42, 127.91, 127.47 (d, *J* = 1.7 Hz), 126.49, 124.98, 124.52, 123.23, 114.84 (d, *J* = 21.1 Hz), 108.12, 58.26, 56.67, 54.30, 38.25, 26.60. ^19^F NMR (376 MHz, CDCl_3_) δ -116.46 (td, *J* = 9.0, 4.7 Hz). HRMS (EI) calculated for [C_26_H_23_FN_2_O]^+^: 398.1794 found: 398.1788. Enantiomeric excess was determined by HPLC with a Chiralpak AD-H column (hexanes/2-propanol = 85/15, 1.0 mL/min, 210 nm); minor enantiomer *t_R_* = 5.5 min, major enantiomer *t_R_* = 11.2 min. [α]_D_^20^ = 131.4 (*c* = 0.3, CHCl_3_).

4.3 Synthesis of (3*R*,4'*S*)-4'-((4-chlorobenzyl)(methyl)amino)-1-methyl-4'*H*-spiro[indoline-3,1'-naphthalen]-2-one (**3c**)

Prepared according to typical procedure from **2c** (85.0 mg, 0.6 mmol), after a flash column chromatography (petroleum ether/ethyl acetate = 8/1) afforded the product **3c** as a pale yellow solid (95.3 mg, 77% yield) with 93% *ee*. Mp: 231-233 ^o^C. ^1^H NMR (400 MHz, CDCl_3_) δ 7.76 (d, *J* = 7.8 Hz, 1H), 7.34 – 7.25 (m, 6H), 7.10 – 7.03 (m, 1H), 6.99 (td, *J* = 7.6, 1.0 Hz, 1H), 6.93 (d, *J* = 7.8 Hz, 1H), 6.87 (dd, *J* = 7.4, 1.2 Hz, 1H), 6.53 (dd, *J* = 7.9, 1.3 Hz, 1H), 6.37 (dd, *J* = 10.2, 3.4 Hz, 1H), 5.75 (dd, *J* = 10.2, 1.6 Hz, 1H), 4.57 (s, 1H), 3.86 (d, *J* = 13.7 Hz, 1H), 3.57 (d, *J* = 13.7 Hz, 1H), 3.29 (s, 3H), 2.37 (s, 3H). ^13^C NMR (101 MHz, CDCl_3_) δ 177.62, 143.52, 138.95, 136.26, 135.56, 135.11, 132.28, 130.02, 129.22, 128.46, 128.25, 127.99, 127.53, 126.53, 124.97, 124.57, 123.28, 108.15, 58.43, 56.69, 54.34, 38.41, 26.65. HRMS (EI) calculated for [C_26_H_23_ClN_2_O]^+^: 414.1499 found: 414.1494. Enantiomeric excess was determined by HPLC with a Chiralpak AD-H column (hexanes/2-propanol = 87/13, 1.0 mL/min, 210 nm); minor enantiomer *t_R_* = 6.0 min, major enantiomer *t_R_* = 14.3 min. [α]_D_^20^ = 120.7 (*c* = 0.3, CHCl_3_).

4.4 Synthesis of (3*R*,4'*S*)-1-methyl-4'-(methyl(4-(trifluoromethyl)benzyl)amino)-4'*H*-spiro[indoline-3,1'-naphthalen]-2-one (**3d**)

Prepared according to typical procedure from **2d** (105.1 mg, 0.6 mmol), after a flash column chromatography (petroleum ether/ethyl acetate = 8/1) afforded the product **3d** as a pale yellow solid (107.6 mg, 80% yield) with 92% *ee*. Mp: 90-92 ^o^C. ^1^H NMR (400 MHz, CDCl_3_) δ 7.78 (d, *J* = 7.8 Hz, 1H), 7.55 (d, *J* = 8.0 Hz, 2H), 7.48 (d, *J* = 8.0 Hz, 2H), 7.29 (qd, *J* = 7.8, 1.3 Hz, 2H), 7.06 (td, *J* = 7.5, 1.4 Hz, 1H), 7.00 (td, *J* = 7.5, 1.0 Hz, 1H), 6.93 (d, *J* = 7.8 Hz, 1H), 6.88 (dd, *J* = 7.4, 1.3 Hz, 1H), 6.54 (dd, *J* = 7.9, 1.4 Hz, 1H), 6.39 (dd, *J* = 10.2, 3.4 Hz, 1H), 5.76 (dd, *J* = 10.2, 1.6 Hz, 1H), 4.64 – 4.55 (m, 1H), 3.96 (d, *J* = 14.1 Hz, 1H), 3.63 (d, *J* = 14.1 Hz, 1H), 3.29 (s, 3H), 2.41 (s, 3H). ^13^C NMR (101 MHz, CDCl_3_) δ 177.56, 144.79, 143.52, 136.10, 135.48, 135.15, 129.25, 128.88 (q, *J* = 32.3 Hz), 128.79, 128.49, 128.12, 127.60, 127.55, 126.58, 125.01 (q, *J* = 3.8 Hz), 124.36 (q, *J* = 272.7 Hz), 124.88, 124.56, 123.29, 108.16, 58.85, 56.64, 54.33, 38.75, 26.63. ^19^F NMR (376 MHz, CDCl_3_) δ -62.22. HRMS (EI) calculated for [C_27_H_23_F_3_N_2_O]^+^: 448.1762 found: 448.1757. Enantiomeric excess was determined by HPLC with a Chiralpak AD-H column (hexanes/2-propanol = 87/13, 1.0 mL/min, 210 nm); minor enantiomer *t_R_* = 5.1 min, major enantiomer *t_R_* = 8.9 min. [α]_D_^20^ = 116.1 (*c* = 0.3, CHCl_3_).

4.5 Synthesis of (3*R*,4'*S*)-1-methyl-4'-morpholino-4'*H*-spiro[indoline-3,1'-naphthalen]-2-one (**3e**)

Prepared according to typical procedure from **2e** (52.3 mg, 0.6 mmol), after a flash column chromatography (petroleum ether/ethyl acetate = 5/1) afforded the product **3e** as a yellow solid (96.9 mg, 95% yield) with 96% *ee*. Mp: 72-74 ^o^C. ^1^H NMR (400 MHz, CDCl_3_) δ 7.76 – 7.69 (m, 1H), 7.30 (td, *J* = 7.7, 1.3 Hz, 1H), 7.25 (td, *J* = 8.0, 7.4, 1.4 Hz, 1H), 7.05 (td, *J* = 7.6, 1.5 Hz, 1H), 6.99 (td, *J* = 7.5, 1.0 Hz, 1H), 6.93 (d, *J* = 7.8 Hz, 1H), 6.88 (dd, *J* = 7.5, 1.3 Hz, 1H), 6.53 (dd, *J* = 7.9, 1.3 Hz, 1H), 6.31 (dd, *J* = 10.2, 3.5 Hz, 1H), 5.72 (dd, *J* = 10.2, 1.6 Hz, 1H), 4.48 (d, *J* = 1.9 Hz, 1H), 3.80-3.72 (m, 2H), 3.72-3.65 (m, 2H), 3.29 (s, 3H), 2.81 – 2.68 (m, 4H). ^13^C NMR (101 MHz, CDCl_3_) δ 177.47, 143.45, 135.44, 135.33, 135.20, 129.25, 128.43, 127.89, 127.45, 127.41, 126.42, 125.36, 124.49, 123.23, 108.12, 67.75, 59.93, 54.21, 49.32, 26.60. HRMS (EI) calculated for [C_22_H_22_N_2_O_2_]^+^: 346.1681 found: 346.1677. Enantiomeric excess was determined by HPLC with a Chiralpak AD-H column (hexanes/2-propanol = 85/15, 1.0 mL/min, 210 nm); minor enantiomer *t_R_* = 12.5 min, major enantiomer *t_R_* = 14.9 min. [α]_D_^20^ = 114.1 (*c* = 0.3, CHCl_3_).

4.6 Synthesis of *tert*-butyl 4-((3*R*,4'*S*)-1-methyl-2-oxo-4'*H*-spiro[indoline-3,1'-naphthalen]-4'-yl)piperazine-1-carboxylate (**3f**)

Prepared according to typical procedure from **2f** (111.7 mg, 0.6 mmol), after a flash column chromatography (petroleum ether/ethyl acetate = 6/1) afforded the product **3f** as a white solid (108.5 mg, 81% yield) with 90% *ee*. Mp: 181-183 ^o^C. ^1^H NMR (400 MHz, CDCl_3_) δ 7.76 – 7.65 (m, 1H), 7.31 (td, *J* = 7.7, 1.3 Hz, 1H), 7.23 (dd, *J* = 7.7, 1.4 Hz, 1H), 7.06 (td, *J* = 7.6, 1.5 Hz, 1H), 7.00 (td, *J* = 7.5, 1.0 Hz, 1H), 6.93 (d, *J* = 7.8 Hz, 1H), 6.88 (dd, *J* = 7.4, 1.3 Hz, 1H), 6.52 (dd, *J* = 7.9, 1.3 Hz, 1H), 6.27 (dd, *J* = 10.2, 3.5 Hz, 1H), 5.69 (dd, *J* = 10.2, 1.6 Hz, 1H), 4.52 (d, *J* = 3.4 Hz, 1H), 3.78 – 3.34 (br, 3H), 3.29 (s, 3H), 3.27 – 3.16 (br, 1H), 2.93-2.48 (m, 4H), 1.44 (s, 9H). ^13^C NMR (101 MHz, CDCl_3_) δ 177.50, 154.72, 143.47, 135.43, 135.39, 135.13, 129.26, 128.46, 127.87, 127.49, 127.44, 126.45, 125.42, 124.52, 123.27, 108.13, 79.25, 59.92, 54.21, 28.37, 26.61. HRMS (EI) calculated for [C_27_H_31_N_3_O_3_]^+^: 445.2365 found: 445.2364. Enantiomeric excess was determined by HPLC with a Chiralpak AD-H column (hexanes/2-propanol = 85/15,, 1.0 mL/min, 210 nm); minor enantiomer *t_R_* = 7.8 min, major enantiomer *t_R_* = 6.8 min. [α]_D_^20^ = 132.0 (*c* = 0.3, CHCl_3_).

4.7 Synthesis of (3*R*,4'*S*)-1-methyl-4'-(phenylamino)-4'*H*-spiro[indoline-3,1'-naphthalen]-2 (**3g**)

Prepared according to typical procedure from **2g** (55.9 mg, 0.6 mmol), after a flash column chromatography (petroleum ether/ethyl acetate = 8/1) afforded the product **3g** as a pale yellow solid (98.7 mg, 93% yield) with 95% *ee*. Mp: 142-145 ^o^C. ^1^H NMR (400 MHz, CDCl_3_) δ 7.50 (dd, *J* = 7.9, 1.4 Hz, 1H), 7.36 (td, *J* = 7.6, 1.5 Hz, 1H), 7.19 (td, *J* = 7.3, 1.1 Hz, 3H), 7.12 – 7.01 (m, 3H), 6.97 – 6.92 (m, 1H), 6.91 – 6.85 (m, 2H), 6.72 (tt, *J* = 7.3, 1.1 Hz, 1H), 6.58 (dd, *J* = 9.6, 4.9 Hz, 1H), 6.53 (dd, *J* = 7.9, 1.4 Hz, 1H), 5.73 (d, *J* = 9.6 Hz, 1H), 5.35 (d, *J* = 6.2 Hz, 1H), 4.93 (d, *J* = 9.9 Hz, 1H), 3.23 (s, 3H). ^13^C NMR (101 MHz, CDCl_3_) δ 176.91, 147.74, 144.18, 137.47, 135.97, 134.19, 131.22, 129.41, 129.14, 128.97, 128.78, 128.02, 127.51, 126.44, 124.91, 123.67, 117.98, 114.53, 108.50, 55.55, 50.71, 27.04. HRMS (EI) calculated for [C_24_H_20_N_2_O]^+^: 352.1576 found: 352.1574. Enantiomeric excess was determined by HPLC with a Chiralpak AD-H column (hexanes/2-propanol = 75/25, 1.0 mL/min, 210 nm); minor enantiomer *t_R_* = 6.6 min, major enantiomer *t_R_* = 10.6 min. [α]_D_^20^ = 134.5 (*c* = 0.3, CHCl_3_).

4.8 Synthesis of (3*R*,4'*S*)-1-methyl-4'-(p-tolylamino)-4'*H*-spiro[indoline-3,1'-naphthalen]-2-one (**3h**)

Prepared according to typical procedure from **2h** (64.3 mg, 0.6 mmol), after a flash column chromatography (petroleum ether/ethyl acetate = 8/1) afforded the product **3h** as a pale yellow solid (98.6 mg, 90% yield) with 92% *ee*. Mp: 131-134 ^o^C. ^1^H NMR (400 MHz, CDCl_3_) δ 7.49 (dd, *J* = 7.7, 1.4 Hz, 1H), 7.37 (td, *J* = 7.6, 1.5 Hz, 1H), 7.23 – 7.17 (m, 1H), 7.13 – 6.99 (m, 5H), 6.95 (d, *J* = 7.8 Hz, 1H), 6.81 (d, *J* = 8.4 Hz, 2H), 6.58 (dd, *J* = 9.6, 4.7 Hz, 1H), 6.52 (dd, *J* = 7.9, 1.3 Hz, 1H), 5.73 (d, *J* = 9.6 Hz, 1H), 5.29 (s, 1H), 4.76 (s, 1H), 3.25 (s, 3H), 2.25 (s, 3H). ^13^C NMR (101 MHz, CDCl_3_) δ 176.91, 145.38, 144.17, 137.55, 135.89, 134.24, 131.39, 129.86, 129.14, 128.89, 128.58, 127.92, 127.40, 127.26, 126.40, 124.88, 123.61, 114.92, 108.41, 55.52, 51.23, 27.01, 20.54. HRMS (EI) calculated for [C_25_H_22_N_2_O]^+^: 366.1732 found: 366.1729. Enantiomeric excess was determined by HPLC with a Chiralpak AD-H column (hexanes/2-propanol = 82/18, 1.0 mL/min, 210 nm); minor enantiomer *t_R_* = 8.5 min, major enantiomer *t_R_* = 16.5 min. [α]_D_^20^ = 148.1 (*c* = 0.3, CHCl_3_).

4.9 Synthesis of (3*R*,4'*S*)-4'-((4-ethylphenyl)amino)-1-methyl-4'*H*-spiro[indoline-3,1'-naphthalen]-2-one (**3i**)

Prepared according to typical procedure from **2i** (72.7 mg, 0.6 mmol), after a flash column chromatography (petroleum ether/ethyl acetate = 8/1) afforded the product **3i** as a white solid (97.3 mg, 85% yield) with 91% *ee*. Mp: 120-122 ^o^C. ^1^H NMR (400 MHz, CDCl_3_) δ 7.55 – 7.49 (m, 1H), 7.37 (td, *J* = 7.6, 1.5 Hz, 1H), 7.23 – 7.18 (m, 1H), 7.13 – 7.02 (m, 5H), 6.95 (dd, *J* = 7.8, 0.9 Hz, 1H), 6.83 (d, *J* = 8.4 Hz, 2H), 6.59 (dd, *J* = 9.7, 4.7 Hz, 1H), 6.52 (dd, *J* = 7.8, 1.3 Hz, 1H), 5.72 (d, *J* = 9.7 Hz, 1H), 5.31 (dd, *J* = 8.8, 4.7 Hz, 1H), 4.77 (d, *J* = 9.6 Hz, 1H), 3.26 (s, 3H), 2.56 (q, *J* = 7.6 Hz, 2H), 1.20 (t, *J* = 7.6 Hz, 3H). ^13^C NMR (101 MHz, CDCl_3_) δ 176.85, 145.46, 144.07, 137.44, 135.76, 134.18, 133.76, 131.27, 129.11, 128.79, 128.58, 128.37, 127.85, 127.32, 126.30, 124.79, 123.51, 114.61, 108.32, 55.40, 50.94, 27.94, 26.92, 15.86. HRMS (EI) calculated for [C_26_H_24_N_2_O]^+^: 380.1889 found: 380.1884. Enantiomeric excess was determined by HPLC with a Chiralpak AD-H column (hexanes/2-propanol = 82/18, 1.0 mL/min, 254 nm); minor enantiomer *t_R_* = 9.0 min, major enantiomer *t_R_* = 18.2 min. [α]_D_^20^ = 152.4 (*c* = 0.3, CHCl_3_).

4.10 Synthesis of (3*R*,4'*S*)-4'-((4-isopropylphenyl)amino)-1-methyl-4'*H*-spiro[indoline-3,1'-naphthalen]-2-one (**3j**)

Prepared according to typical procedure from **2j** (81.1 mg, 0.6 mmol), after a flash column chromatography (petroleum ether/ethyl acetate = 8/1) afforded the product **3j** as a yellow solid (103.3 mg, 87% yield) with 92% *ee*. Mp: 64-66 ^o^C. ^1^H NMR (400 MHz, CDCl_3_) δ 7.51 (dd, *J* = 7.7, 1.4 Hz, 1H), 7.36 (td, *J* = 7.6, 1.5 Hz, 1H), 7.21 (td, *J* = 7.5, 1.4 Hz, 1H), 7.13 – 7.01 (m, 5H), 6.95 (dd, *J* = 7.9, 1.0 Hz, 1H), 6.83 (d, *J* = 8.5 Hz, 2H), 6.59 (dd, *J* = 9.7, 4.7 Hz, 1H), 6.52 (dd, *J* = 7.9, 1.3 Hz, 1H), 5.72 (d, *J* = 9.7 Hz, 1H), 5.31 (d, *J* = 4.7 Hz, 1H), 4.70 (s, 1H), 3.25 (s, 3H), 2.82 (hept, *J* = 7.0 Hz, 1H), 1.22 (d, *J* = 6.9 Hz, 6H). ^13^C NMR (101 MHz, CDCl_3_) δ 176.84, 145.40, 144.04, 138.42, 137.37, 135.76, 134.16, 131.21, 129.18, 128.79, 128.35, 127.85, 127.32, 127.12, 126.27, 124.77, 123.50, 114.40, 108.32, 55.38, 50.80, 33.14, 26.90, 24.20. HRMS (EI) calculated for [C_27_H_26_N_2_O]^+^: 394.2045 found: 394.2041. Enantiomeric excess was determined by HPLC with a Chiralpak AD-H column (hexanes/2-propanol = 82/18, 1.0 mL/min, 254 nm); minor enantiomer *t_R_* = 8.2 min, major enantiomer *t_R_* = 16.1 min. [α]_D_^20^ = 143.1 (*c* = 0.3, CHCl_3_).

4.11 Synthesis of (3*R*,4'*S*)-4'-((4-(tert-butyl)phenyl)amino)-1-methyl-4'*H*-spiro[indoline-3,1'-naphthalen]-2-one (**3k**)

Prepared according to typical procedure from **2k** (89.5 mg, 0.6 mmol), after a flash column chromatography (petroleum ether/ethyl acetate = 8/1) afforded the product **3k** as a pale yellow solid (96.9 mg, 79% yield) with 90% *ee*. Mp: 86-88 ^o^C. ^1^H NMR (400 MHz, CDCl_3_) δ 7.53 (dd, *J* = 7.8, 1.4 Hz, 1H), 7.36 (td, *J* = 7.6, 1.5 Hz, 1H), 7.24 – 7.18 (m, 3H), 7.12 – 7.02 (m, 3H), 6.94 (d, *J* = 7.8 Hz, 1H), 6.83 (d, *J* = 8.7 Hz, 2H), 6.62 – 6.56 (m, 1H), 6.52 (dd, *J* = 7.9, 1.3 Hz, 1H), 5.71 (d, *J* = 9.6 Hz, 1H), 5.37 – 5.27 (m, 1H), 4.78 (d, *J* = 10.0 Hz, 1H), 3.24 (s, 3H), 1.29 (s, 9H). ^13^C NMR (101 MHz, CDCl_3_) δ 176.81, 145.09, 144.05, 140.50, 137.43, 135.76, 134.17, 131.22, 129.19, 128.78, 128.27, 127.87, 127.31, 126.25, 126.03, 124.77, 123.48, 113.86, 108.30, 55.36, 50.56, 33.84, 31.54, 26.89. HRMS (EI) calculated for [C_28_H_28_N_2_O]^+^: 408.2202 found: 408.2195. Enantiomeric excess was determined by HPLC with a Chiralpak AD-H column (hexanes/2-propanol = 82/18, 1.0 mL/min, 210 nm); minor enantiomer *t_R_* = 8.2 min, major enantiomer *t_R_* = 18.1 min. [α]_D_^20^ = 148.9 (*c* = 0.3, CHCl_3_).

4.12 Synthesis of (3*R*,4'*S*)-4'-([1,1'-biphenyl]-4-ylamino)-1-methyl-4'*H*-spiro[indoline-3,1'-naphthalen]-2-one (**3l**)

Prepared according to typical procedure from **2l** (101.5 mg, 0.6 mmol), after a flash column chromatography (petroleum ether/ethyl acetate = 8/1) afforded the product **3l** as a pale yellow solid (94.7 mg, 74% yield) with 92% *ee*. Mp: 95-97 ^o^C. ^1^H NMR (400 MHz, CDCl_3_) δ 7.55 (ddd, *J* = 8.7, 7.4, 1.4 Hz, 3H), 7.48 (d, *J* = 8.6 Hz, 2H), 7.38 (dd, *J* = 8.6, 6.8 Hz, 3H), 7.26 – 7.19 (m, 2H), 7.17 – 7.04 (m, 3H), 7.02 – 6.93 (m, 3H), 6.62 (dd, *J* = 9.6, 4.9 Hz, 1H), 6.54 (dd, *J* = 7.9, 1.3 Hz, 1H), 5.77 (d, *J* = 9.6 Hz, 1H), 5.40 (dd, *J* = 10.2, 4.9 Hz, 1H), 5.06 (d, *J* = 10.5 Hz, 1H), 3.26 (s, 3H). ^13^C NMR (101 MHz, CDCl_3_) δ 176.80, 147.05, 144.10, 141.26, 137.28, 135.90, 134.04, 131.02, 130.64, 129.05, 128.87, 128.59, 127.99, 127.97, 127.46, 126.36, 126.26, 125.99, 124.84, 123.59, 114.51, 108.39, 55.49, 50.59, 26.97. HRMS (EI) calculated for [C_30_H_24_N_2_O]^+^: 428.1889 found: 428.1885. Enantiomeric excess was determined by HPLC with a Chiralpak OD-H column (hexanes/2-propanol = 85/15, 1.0 mL/min, 210 nm); minor enantiomer *t_R_* = 18.3 min, major enantiomer *t_R_* = 14.1 min. [α]_D_^20^ = 112.3 (*c* = 0.3, CHCl_3_).

4.13 Synthesis of (3*R*,4'*S*)-4'-((4-chlorophenyl)amino)-1-methyl-4'*H*-spiro[indoline-3,1'-naphthalen]-2-one (**3m**)

Prepared according to typical procedure from **2m** (76.6 mg, 0.6 mmol), after a flash column chromatography (petroleum ether/ethyl acetate = 8/1) afforded the product **3m** as a pale yellow solid (91.5 mg, 79% yield) with 94% *ee*. Mp: 136-138 ^o^C. ^1^H NMR (400 MHz, CDCl_3_) δ 7.46 (dd, *J* = 7.6, 1.4 Hz, 1H), 7.39 (td, *J* = 7.7, 1.4 Hz, 1H), 7.22 (td, *J* = 7.5, 1.3 Hz, 1H), 7.17 – 7.09 (m, 3H), 7.09 – 7.04 (m, 2H), 6.97 (dt, *J* = 7.8, 0.8 Hz, 1H), 6.84 – 6.78 (m, 2H), 6.57 (dd, *J* = 9.6, 4.9 Hz, 1H), 6.53 (dd, *J* = 7.9, 1.3 Hz, 1H), 5.77 (d, *J* = 9.6 Hz, 1H), 5.28 (dd, *J* = 10.6, 4.9 Hz, 1H), 5.05 (d, *J* = 10.7 Hz, 1H), 3.26 (s, 3H). ^13^C NMR (101 MHz, CDCl_3_) δ 176.75, 146.26, 144.07, 137.09, 135.94, 133.90, 130.88, 129.22, 129.10, 128.93, 128.87, 127.96, 127.50, 126.40, 124.83, 123.65, 122.31, 115.69, 108.42, 55.53, 51.02, 26.97. HRMS (EI) calculated for [C_24_H_19_ClN_2_O]^+^: 386.1186 found: 386.1182. Enantiomeric excess was determined by HPLC with a Chiralpak AD-H column (hexanes/2-propanol = 82/18, 1.0 mL/min, 254 nm); minor enantiomer *t_R_* = 8.6 min, major enantiomer *t_R_* = 15.6 min. [α]_D_^20^ = 109.6 (*c* = 0.3, CHCl_3_).

4.14 Synthesis of (3*R*,4'*S*)-4'-((4-fluorophenyl)amino)-1-methyl-4'*H*-spiro[indoline-3,1'-naphthalen]-2-one (**3n**)

Prepared according to typical procedure from **2n** (66.7 mg, 0.6 mmol), after a flash column chromatography (petroleum ether/ethyl acetate = 8/1) afforded the product **3n** as a white solid (102.9 mg, 93% yield) with 92% *ee*. Mp: 136-138 ^o^C. ^1^H NMR (400 MHz, CDCl_3_) δ 7.47 – 7.41 (m, 1H), 7.38 (td, *J* = 7.7, 1.4 Hz, 1H), 7.19 (td, *J* = 7.5, 1.3 Hz, 1H), 7.13 – 7.08 (m, 1H), 7.05 (ddd, *J* = 6.3, 4.7, 3.2 Hz, 2H), 6.96 (d, *J* = 7.8 Hz, 1H), 6.90 (t, *J* = 8.7 Hz, 2H), 6.82 (dd, *J* = 9.0, 4.5 Hz, 2H), 6.58 (dd, *J* = 9.6, 4.9 Hz, 1H), 6.53 (d, *J* = 7.8 Hz, 1H), 5.76 (d, *J* = 9.5 Hz, 1H), 5.22 (dd, *J* = 9.3, 4.9 Hz, 1H), 4.88 (d, *J* = 9.8 Hz, 1H), 3.24 (s, 3H). ^13^C NMR (101 MHz, CDCl_3_) δ 176.72, 156.14 (d, *J* = 235.6 Hz), 144.05, 143.96 (d, *J* = 2.1 Hz), 137.18, 135.94, 133.94, 131.20, 129.09, 128.90, 128.87, 127.79, 127.38, 126.37, 124.78, 123.58, 116.01 (d, *J* = 7.5 Hz), 115.63 (d, *J* = 22.4 Hz), 108.37, 55.53, 51.95, 26.92. ^19^F NMR (376 MHz, CDCl_3_) δ -126.91. HRMS (EI) calculated for [C_24_H_19_FN_2_O]^+^: 370.1481 found: 370.1476. Enantiomeric excess was determined by HPLC with a Chiralpak AD-H column (hexanes/2-propanol = 85/15, 1.0 mL/min, 315 nm); minor enantiomer *t_R_* = 7.9 min, major enantiomer *t_R_* = 13.1 min. [α]_D_^20^ = 146.3 (*c* = 0.3, CHCl_3_).

4.15 Synthesis of (3*R*,4'*S*)-1-methyl-4'-((4-(trifluoromethoxy)phenyl)amino)-4'*H*-spiro[indoline-3,1'-naphthalen]-2-one (**3o**)

Prepared according to typical procedure from **2o** (106.3 mg, 0.6 mmol), after a flash column chromatography (petroleum ether/ethyl acetate = 8/1) afforded the product **3o** as a white solid (107.9 mg, 82% yield) with 92% *ee*. Mp: 140-143 ^o^C. ^1^H NMR (400 MHz, CDCl_3_) δ 7.48 (dd, *J* = 7.7, 1.4 Hz, 1H), 7.39 (td, *J* = 7.7, 1.4 Hz, 1H), 7.26-7.20 (mo, 1H), 7.15 – 7.02 (m, 5H), 6.97 (d, *J* = 7.8 Hz, 1H), 6.84 (d, *J* = 8.9 Hz, 2H), 6.58 (dd, *J* = 9.6, 4.9 Hz, 1H), 6.53 (dd, *J* = 7.9, 1.3 Hz, 1H), 5.78 (d, *J* = 9.6 Hz, 1H), 5.30 (dd, *J* = 10.6, 4.9 Hz, 1H), 5.12 (d, *J* = 10.6 Hz, 1H), 3.26 (s, 3H). ^13^C NMR (101 MHz, CDCl_3_) δ 146.34, 144.09, 140.77, 136.98, 136.04, 133.86, 130.82, 129.40, 128.98, 128.96, 128.01, 127.56, 126.41, 124.85, 123.67, 122.39, 120.70 (q, *J* = 256.0 Hz), 114.77, 108.45, 55.57, 51.06, 26.98. ^19^F NMR (376 MHz, CDCl_3_) δ -58.41. HRMS (EI) calculated for [C_25_H_19_F_3_N_2_O_2_]^+^: 436.1399 found: 436.1396. Enantiomeric excess was determined by HPLC with a Chiralpak AD-H column (hexanes/2-propanol = 82/18, 1.0 mL/min, 254 nm); minor enantiomer *t_R_* = 6.9 min, major enantiomer *t_R_* = 13.1 min. [α]_D_^20^ = 132.1 (*c* = 0.3, CHCl_3_).

4.16 Synthesis of 4-(((3*R*,4'*S*)-1-methyl-2-oxo-4'*H*-spiro[indoline-3,1'-naphthalen]-4'-yl)amino)benzonitrile (**3p**)

Prepared according to typical procedure from **2p** (70.9 mg, 0.6 mmol), after a flash column chromatography (petroleum ether/ethyl acetate = 5/1) afforded the product **3p** as a white solid (52.7 mg, 47% yield) with 94% *ee*. Mp: 199-200 ^o^C. ^1^H NMR (400 MHz, CDCl_3_) δ 7.43 (dt, *J* = 13.2, 7.6 Hz, 4H), 7.26 (d, *J* = 7.1 Hz, 1H), 7.18 – 7.06 (m, 3H), 7.00 (d, *J* = 7.8 Hz, 1H), 6.83 (d, *J* = 8.4 Hz, 2H), 6.54 (dd, *J* = 9.0, 5.5 Hz, 2H), 5.84 (d, *J* = 9.6 Hz, 1H), 5.78 (d, *J* = 10.2 Hz, 1H), 5.41 (dd, *J* = 10.2, 5.2 Hz, 1H), 3.26 (s, 3H). ^13^C NMR (101 MHz, CDCl_3_) δ 176.57, 150.68, 144.03, 136.46, 136.17, 133.80, 133.46, 130.28, 130.00, 129.10, 128.66, 128.23, 127.82, 126.47, 124.85, 123.79, 120.48, 113.07, 108.57, 98.67, 55.61, 49.73, 27.02. HRMS (EI) calculated for [C_25_H_19_N_3_O]^+^: 377.1528 found: 377.1524. Enantiomeric excess was determined by HPLC with a Chiralpak OD-H column (hexanes/2-propanol = 85/15, 1.0 mL/min, 210 nm); minor enantiomer *t_R_* = 14.1 min, major enantiomer *t_R_* = 16.8 min. [α]_D_^20^ = 124.8 (*c* = 0.3, CHCl_3_).

4.17 Synthesis of (3*R*,4'*S*)-4'-((3-fluorophenyl)amino)-1-methyl-4'*H*-spiro[indoline-3,1'-naphthalen]-2-one (**3q**)

Prepared according to typical procedure from **2q** (111.1 mg, 0.6 mmol), after a flash column chromatography (petroleum ether/ethyl acetate = 8/1) afforded the product **3q** as a white solid (102.7 mg, 92% yield) with 91% *ee*. Mp: 110-111 ^o^C. ^1^H NMR (400 MHz, CDCl_3_) δ 7.50 (dd, *J* = 7.8, 1.4 Hz, 1H), 7.39 (td, *J* = 7.6, 1.4 Hz, 1H), 7.26 – 7.20 (m, 1H), 7.15 – 7.05 (m, 4H), 6.97 (d, *J* = 7.8 Hz, 1H), 6.64 – 6.55 (m, 3H), 6.53 (dd, *J* = 7.9, 1.3 Hz, 1H), 6.44 – 6.37 (m, 1H), 5.78 (d, *J* = 9.6 Hz, 1H), 5.30 (dd, *J* = 10.6, 4.9 Hz, 1H), 5.17 (d, *J* = 10.6 Hz, 1H), 3.26 (s, 3H). ^13^C NMR (101 MHz, CDCl_3_) δ 176.79, 164.20 (d, *J* = 242.9 Hz), 149.50 (d, *J* = 10.6 Hz), 144.15, 137.09, 136.05, 133.93, 130.81, 130.46, 130.36, 129.33, 129.01 (d, *J* = 1.8 Hz), 128.09, 127.60, 126.44, 124.91, 123.70, 110.18 (d, *J* = 2.2 Hz), 108.49, 104.19 (d, *J* = 21.6 Hz), 100.77 (d, *J* = 25.0 Hz), 55.57, 50.64, 27.03. ^19^F NMR (376 MHz, CDCl_3_) δ -112.98 (dt, *J* = 12.1, 7.7 Hz). HRMS (EI) calculated for [C_24_H_19_FN_2_O]^+^: 370.1481 found: 370.1473. Enantiomeric excess was determined by HPLC with a Chiralpak IA column (hexanes/2-propanol = 90/10, 1.0 mL/min, 254 nm); minor enantiomer *t_R_* = 7.7 min, major enantiomer *t_R_* = 9.6 min. [α]_D_^20^ = 163.7 (*c* = 0.3, CHCl_3_).

4.18 Synthesis of (3*R*,4'*S*)-4'-((3-chlorophenyl)amino)-1-methyl-4'*H*-spiro[indoline-3,1'-naphthalen]-2-one (**3r**)

Prepared according to typical procedure from **2r** (127.6 mg, 0.6 mmol), after a flash column chromatography (petroleum ether/ethyl acetate = 8/1) afforded the product **3r** as a white solid (82.2 mg, 71% yield) with 92% *ee*. Mp: 148-151 ^o^C. ^1^H NMR (400 MHz, CDCl_3_) δ 7.47 (dd, *J* = 7.8, 1.5 Hz, 1H), 7.38 (td, *J* = 7.7, 1.5 Hz, 1H), 7.21 (td, *J* = 7.5, 1.3 Hz, 1H), 7.15-7.02 (m, 4H), 6.96 (d, *J* = 7.8 Hz, 1H), 6.87 (t, *J* = 2.1 Hz, 1H), 6.72 (ddd, *J* = 8.2, 2.3, 1.0 Hz, 1H), 6.67 (ddd, *J* = 7.8, 2.0, 0.9 Hz, 1H), 6.61 – 6.49 (m, 2H), 5.77 (d, *J* = 9.6 Hz, 1H), 5.30 (dd, *J* = 10.6, 4.9 Hz, 1H), 5.16 (d, *J* = 10.6 Hz, 1H), 3.24 (s, 3H). ^13^C NMR (101 MHz, CDCl_3_) δ 176.64, 148.80, 144.03, 136.95, 135.99, 134.95, 133.77, 130.68, 130.23, 129.33, 128.92, 128.89, 127.99, 127.51, 126.34, 124.79, 123.61, 117.51, 113.71, 112.57, 108.41, 55.49, 50.48, 26.93. HRMS (EI) calculated for [C_24_H_19_ClN_2_O]^+^: 386.1186 found: 386.1183. Enantiomeric excess was determined by HPLC with a Chiralpak AD-H column (hexanes/2-propanol = 90/10, 1.0 mL/min, 254 nm); minor enantiomer *t_R_* = 8.3 min, major enantiomer *t_R_* = 10.4 min. [α]_D_^20^ = 152.5 (*c* = 0.3, CHCl_3_).

4.19 Synthesis of (3*R*,4'*S*)-4'-((3-(tert-butyl)phenyl)amino)-1-methyl-4'*H*-spiro[indoline-3,1'-naphthalen]-2-one (**3s**)

Prepared according to typical procedure from **2s** (111.1 mg, 0.6 mmol), after a flash column chromatography (petroleum ether/ethyl acetate = 8/1) afforded the product **3s** as a pale yellow solid (80.7 mg, 66% yield) with 91% *ee*. Mp: 140-142 ^o^C. ^1^H NMR (400 MHz, CDCl_3_) δ 7.53 (d, *J* = 7.7 Hz, 1H), 7.36 (td, *J* = 7.6, 1.7 Hz, 1H), 7.22 – 7.12 (m, 2H), 7.12 – 7.02 (m, 3H), 6.94 (d, *J* = 7.8 Hz, 1H), 6.87 (d, *J* = 2.2 Hz, 1H), 6.78 (d, *J* = 7.8 Hz, 1H), 6.74 (d, *J* = 7.9 Hz, 1H), 6.59 (dd, *J* = 9.7, 4.7 Hz, 1H), 6.53 (d, *J* = 7.8 Hz, 1H), 5.72 (d, *J* = 9.8 Hz, 1H), 5.42 – 5.33 (m, 1H), 4.82 (d, *J* = 9.8 Hz, 1H), 3.24 (s, 3H), 1.31 (s, 9H). ^13^C NMR (101 MHz, CDCl_3_) δ 176.78, 152.28, 147.24, 144.03, 137.37, 135.72, 134.12, 130.98, 129.08, 128.84, 128.78, 128.32, 127.84, 127.31, 126.25, 124.75, 123.48, 115.10, 112.03, 110.77, 108.30, 55.32, 50.36, 34.56, 31.32, 26.88. HRMS (EI) calculated for [C_28_H_28_N_2_O]^+^: 408.2202 found: 408.2197. Enantiomeric excess was determined by HPLC with a Chiralpak AD-H column (hexanes/2-propanol = 90/10, 1.0 mL/min, 254 nm); minor enantiomer *t_R_* = 5.6 min, major enantiomer *t_R_* = 6.9 min. [α]_D_^20^ = 129.6 (*c* = 0.3, CHCl_3_).

4.20 Synthesis of (3*R*,4'*S*)-4'-((3-methoxyphenyl)amino)-1-methyl-4'*H*-spiro[indoline-3,1'-naphthalen]-2-one (**3t**)

Prepared according to typical procedure from **2t** (73.9 mg, 0.6 mmol), after a flash column chromatography (petroleum ether/ethyl acetate = 6/1) afforded the product **3t** as a yellow solid (98.1 mg, 86% yield) with 94% *ee*. Mp: 84-87 ^o^C. ^1^H NMR (400 MHz, CDCl_3_) δ 7.53 (dd, *J* = 7.7, 1.4 Hz, 1H), 7.37 (td, *J* = 7.6, 1.5 Hz, 1H), 7.23 – 7.19 (m, 1H), 7.14 – 7.03 (m, 4H), 6.96 (d, *J* = 7.8 Hz, 1H), 6.59 (dd, *J* = 9.6, 4.8 Hz, 1H), 6.55 – 6.47 (m, 2H), 6.44 (t, *J* = 2.3 Hz, 1H), 6.30 (ddd, *J* = 8.1, 2.4, 0.8 Hz, 1H), 5.74 (d, *J* = 9.6 Hz, 1H), 5.33 (dd, *J* = 10.0, 4.8 Hz, 1H), 4.95 (d, *J* = 10.4 Hz, 1H), 3.78 (s, 3H), 3.25 (s, 3H). ^13^C NMR (101 MHz, CDCl_3_) δ 176.78, 160.83, 148.96, 144.05, 137.26, 135.81, 134.03, 130.96, 130.01, 129.04, 128.83, 128.64, 127.93, 127.39, 126.28, 124.78, 123.53, 108.34, 107.18, 103.11, 100.08, 55.39, 55.08, 50.45, 26.91. HRMS (EI) calculated for [C_25_H_22_N_2_O_2_]^+^: 382.1681 found: 382.1676. Enantiomeric excess was determined by HPLC with a Chiralpak AD-H column (hexanes/2-propanol = 83/17, 1.0 mL/min, 254 nm); minor enantiomer *t_R_* = 12.0 min, major enantiomer *t_R_* = 14.9 min. [α]_D_^20^ = 137.4 (*c* = 0.3, CHCl_3_).

4.21 Synthesis of (3*R*,4'*S*)-1-methyl-4'-((3-(methylthio)phenyl)amino)-4'*H*-spiro[indoline-3,1'-naphthalen]-2-one (**3u**)

Prepared according to typical procedure from **2u** (83.5 mg, 0.6 mmol), after a flash column chromatography (petroleum ether/ethyl acetate = 6/1) afforded the product **3u** as a yellow solid (92.2 mg, 77% yield) with 92% *ee*. Mp: 81-84 ^o^C. ^1^H NMR (400 MHz, CDCl_3_) δ 7.48 (dd, *J* = 7.7, 1.4 Hz, 1H), 7.38 (td, *J* = 7.6, 1.5 Hz, 1H), 7.19 (d, *J* = 1.3 Hz, 1H), 7.15 – 7.02 (m, 4H), 6.99 – 6.93 (m, 1H), 6.79 (t, *J* = 2.0 Hz, 1H), 6.71-6.62 (m, 2H), 6.58 (dd, *J* = 9.7, 4.8 Hz, 1H), 6.53 (dd, *J* = 7.8, 1.3 Hz, 1H), 5.76 (d, *J* = 9.7 Hz, 1H), 5.33 (d, *J* = 4.9 Hz, 1H), 5.06 (s, 1H), 3.25 (s, 3H), 2.45 (s, 3H). ^13^C NMR (101 MHz, CDCl_3_) δ 176.70, 147.67, 144.02, 139.24, 136.97, 135.89, 133.89, 130.79, 129.59, 129.01, 128.86, 127.92, 127.45, 126.28, 124.77, 123.56, 116.16, 112.40, 111.44, 108.38, 55.43, 50.62, 26.91, 15.75. HRMS (EI) calculated for [C_25_H_22_N_2_OS]^+^: 398.1453 found: 398.1448. Enantiomeric excess was determined by HPLC with a Chiralpak AD-H column (hexanes/2-propanol = 82/18, 1.0 mL/min, 210 nm); minor enantiomer *t_R_* = 9.7 min, major enantiomer *t_R_* = 12.0 min. [α]_D_^20^ = 119.2 (*c* = 0.3, CHCl_3_).

4.22 Synthesis of (3*R*,4'*S*)-4'-([1,1'-biphenyl]-3-ylamino)-1-methyl-4'*H*-spiro[indoline-3,1'-naphthalen]-2-one (**3v**)

Prepared according to typical procedure from **2v** (89.5 mg, 0.6 mmol), after a flash column chromatography (petroleum ether/ethyl acetate = 8/1) afforded the product **3v** as a pale yellow solid (101.7 mg, 79% yield) with 93% *ee*. Mp: 94-97 ^o^C. ^1^H NMR (400 MHz, CDCl_3_) δ 7.60 (dt, *J* = 6.9, 1.4 Hz, 2H), 7.57 – 7.51 (m, 1H), 7.43 – 7.19 (m, 6H), 7.13 – 7.03 (m, 4H), 6.99 – 6.92 (m, 2H), 6.91 – 6.86 (m, 1H), 6.62 (ddt, *J* = 9.7, 4.9, 1.1 Hz, 1H), 6.54 (dd, *J* = 7.8, 1.6 Hz, 1H), 5.75 (d, *J* = 9.6 Hz, 1H), 5.42 (dd, *J* = 10.4, 4.8 Hz, 1H), 5.06 (d, *J* = 10.5 Hz, 1H), 3.24 (s, 3H). ^13^C NMR (101 MHz, CDCl_3_) δ 176.74, 147.94, 144.06, 142.40, 141.73, 137.29, 135.89, 134.00, 131.01, 129.64, 129.04, 128.84, 128.79, 128.51, 127.93, 127.40, 127.17, 127.04, 126.31, 124.79, 123.53, 116.98, 113.23, 113.18, 108.35, 55.44, 50.60, 26.91. HRMS (EI) calculated for [C_30_H_24_N_2_O]^+^: 428.1889 found: 428.1884. Enantiomeric excess was determined by HPLC with a Chiralpak AD-H column (hexanes/2-propanol = 82/18, 1.0 mL/min, 210 nm); minor enantiomer *t_R_* = 9.7 min, major enantiomer *t_R_* = 13.2 min. [α]_D_^20^ = 106.1 (*c* = 0.3, CHCl_3_).

4.23 Synthesis of (3*R*,4'*S*)-4'-((3,5-dimethylphenyl)amino)-1-methyl-4'*H*-spiro[indoline-3,1'-naphthalen]-2-one (**3w**)

Prepared according to typical procedure from **2w** (72.7 mg, 0.6 mmol), after a flash column chromatography (petroleum ether/ethyl acetate = 8/1) afforded the product **3w** as a yellow solid (106.8 mg, 94% yield) with 92% *ee*. Mp: 133-134 ^o^C. ^1^H NMR (400 MHz, CDCl_3_) δ 7.52 (dd, *J* = 7.8, 1.5 Hz, 1H), 7.37 (td, J = 7.6, 1.6 Hz, 1H), 7.24 – 7.19 (m, 1H), 7.13 – 7.03 (m, 3H), 6.96 (d, *J* = 7.8 Hz, 1H), 6.58 (dd, *J* = 9.7, 4.7 Hz, 1H), 6.55 – 6.48 (m, 3H), 6.40 (s, 1H), 5.72 (d, *J* = 9.6 Hz, 1H), 5.34 (dd, *J* = 10.5, 4.7 Hz, 1H), 4.78 (d, *J* = 10.5 Hz, 1H), 3.26 (s, 3H), 2.26 (s, 6H). ^13^C NMR (101 MHz, CDCl_3_) δ 176.86, 147.59, 144.06, 138.87, 137.48, 135.75, 134.16, 131.19, 129.10, 128.79, 128.31, 127.90, 127.31, 126.26, 124.79, 123.51, 119.85, 112.17, 108.32, 55.38, 50.41, 26.92, 21.49. HRMS (EI) calculated for [C_26_H_24_N_2_O]^+^: 380.1889 found: 380.1885. Enantiomeric excess was determined by HPLC with a Chiralpak AD-H column (hexanes/2-propanol = 85/15, 1.0 mL/min, 254 nm); minor enantiomer *t_R_* = 6.4 min, major enantiomer *t_R_* = 9.0 min. [α]_D_^20^ = 122.3 (*c* = 0.3, CHCl_3_).

4.24 Synthesis of (3*R*,4'*S*)-1-methyl-4'-((3,4,5-trimethylphenyl)amino)-4'*H*-spiro[indoline-3,1'-naphthalen]-2-one (**3x**)

Prepared according to typical procedure from **2x** (81.2 mg, 0.6 mmol), after a flash column chromatography (petroleum ether/ethyl acetate = 8/1) afforded the product **3x** as a yellow solid (114.2 mg, 97% yield) with 88% *ee*. Mp: 76-78 ^o^C. ^1^H NMR (400 MHz, CDCl_3_) δ 7.53 (dd, *J* = 7.7, 1.4 Hz, 1H), 7.37 (td, *J* = 7.6, 1.6 Hz, 1H), 7.24 – 7.18 (m, 1H), 7.14 – 7.01 (m, 3H), 6.96 (d, *J* = 7.8 Hz, 1H), 6.66-6.56 (m, 3H), 6.52 (dd, *J* = 7.9, 1.3 Hz, 1H), 5.71 (d, *J* = 9.7 Hz, 1H), 5.30 (s, 1H), 4.61 (s, 1H), 3.26 (s, 3H), 2.24 (s, 6H), 2.09 (s, 3H). ^13^C NMR (101 MHz, CDCl_3_) δ 176.91, 144.94, 144.08, 137.58, 137.21, 135.69, 134.27, 131.33, 129.17, 128.77, 128.08, 127.87, 127.27, 126.25, 124.80, 124.44, 123.49, 113.96, 108.30, 55.37, 50.67, 26.93, 20.81, 14.47. HRMS (EI) calculated for [C_27_H_26_N_2_O]^+^: 394.2045 found: 394.2041. Enantiomeric excess was determined by HPLC with a Chiralpak AD-H column (hexanes/2-propanol = 80/20, 1.0 mL/min, 254 nm); minor enantiomer *t_R_* = 7.2 min, major enantiomer *t_R_* = 9.1 min. [α]_D_^20^ = 133.2 (*c* = 0.3, CHCl_3_).

4.25 Synthesis of (3*R*,4'*S*)-1-methyl-4'-(naphthalen-1-ylamino)-4'*H*-spiro[indoline-3,1'-naphthalen]-2-one (**3y**)

Prepared according to typical procedure from **2y** (85.9 mg, 0.6 mmol), after a flash column chromatography (petroleum ether/ethyl acetate = 8/1) afforded the product **3y** as a pink solid (63.6 mg, 53% yield) with 90% *ee*. Mp: 191-193 ^o^C. ^1^H NMR (400 MHz, CDCl_3_) δ 8.06 (dd, *J* = 6.5, 3.4 Hz, 1H), 7.76 (dd, *J* = 6.3, 3.3 Hz, 1H), 7.56 (dd, *J* = 7.7, 1.4 Hz, 1H), 7.46-7.36 (m, 4H), 7.27 – 7.20 (m, 2H), 7.17 – 7.05 (m, 4H), 6.97 (d, *J* = 7.6 Hz, 1H), 6.73 (dd, *J* = 9.6, 5.0 Hz, 1H), 6.56 (d, *J* = 7.9 Hz, 1H), 5.90 (s, 1H), 5.81 (d, *J* = 9.6 Hz, 1H), 5.61 (dd, *J* = 10.0, 5.0 Hz, 1H), 3.28 (s, 3H). ^13^C NMR (101 MHz, CDCl_3_) δ 176.76, 144.23, 143.12, 137.51, 136.21, 134.73, 133.95, 130.98, 129.28, 129.21, 128.91, 128.26, 128.05, 127.44, 126.36, 126.25, 125.70, 124.89, 124.74, 124.30, 123.60, 121.22, 117.46, 108.41, 105.08, 55.68, 50.40, 27.07. HRMS (ESI) calculated for C_28_H_23_N_2_O: 403.1805 (M+H^+^), found: 403.1800. Enantiomeric excess was determined by HPLC with a Chiralpak AD-H column (hexanes/2-propanol = 82/18, 1.0 mL/min, 254 nm); minor enantiomer *t_R_* = 6.4 min, major enantiomer *t_R_* = 6.9 min. [α]_D_^20^ = 161.7 (*c* = 0.3, CHCl_3_).

4.26 Synthesis of (3*R*,4'*S*)-1-methyl-4'-(naphthalen-2-ylamino)-4'*H*-spiro[indoline-3,1'-naphthalen]-2-one (**3z**)

Prepared according to typical procedure from **2z** (85.9 mg, 0.6 mmol), after a flash column chromatography (petroleum ether/ethyl acetate = 8/1) afforded the product **3z** as a yellow solid (82.2 mg, 68% yield) with 90% *ee*. Mp: 102-105 ^o^C. ^1^H NMR (400 MHz, CDCl_3_) δ 7.73 – 7.63 (m, 3H), 7.55 (dd, *J* = 7.7, 1.4 Hz, 1H), 7.43 – 7.32 (m, 2H), 7.25-7.17 (m, 3H), 7.16 – 7.03 (m, 4H), 6.96 (d, *J* = 7.8 Hz, 1H), 6.66 (dd, *J* = 9.6, 4.9 Hz, 1H), 6.54 (dd, *J* = 7.9, 1.3 Hz, 1H), 5.78 (d, *J* = 9.6 Hz, 1H), 5.50 (dd, *J* = 10.2, 4.9 Hz, 1H), 5.18 (d, *J* = 10.2 Hz, 1H), 3.26 (s, 3H). ^13^C NMR (101 MHz, CDCl_3_) δ 176.84, 145.34, 144.09, 137.28, 135.90, 135.18, 134.02, 130.97, 129.06, 128.99, 128.94, 128.88, 127.98, 127.82, 127.61, 127.47, 126.36, 126.20, 125.88, 124.84, 123.60, 122.00, 119.05, 108.39, 106.19, 55.51, 50.54, 26.96. HRMS (EI) calculated for [C_28_H_22_N_2_O]^+^: 402.1732 found: 402.1726. Enantiomeric excess was determined by HPLC with a Chiralpak AD-H column (hexanes/2-propanol = 83/17, 1.0 mL/min, 254 nm); minor enantiomer *t_R_* = 13.5 min, major enantiomer *t_R_* = 17.4 min. [α]_D_^20^ = 69.4 (*c* = 0.3, CHCl_3_).

4.27 Synthesis of (3*R*,4'*S*)-4'-(benzyl(methyl)amino)-1,6-dimethyl-4'*H*-spiro[indoline-3,1'-naphthalen]-2-one (**3aa**)

Prepared according to typical procedure from **1aa** (126.5 mg, 0.3 mmol), after a flash column chromatography (petroleum ether/ethyl acetate = 8/1) afforded the product **3aa** as a pale yellow solid (98.5 mg, 83% yield) with 92% *ee*. Mp: 168-171 ^o^C. ^1^H NMR (400 MHz, CDCl_3_) δ 7.77 (d, *J* = 7.8 Hz, 1H), 7.41 (d, *J* = 7.1 Hz, 2H), 7.38 – 7.19 (m, 4H), 7.04 (td, *J* = 7.6, 1.4 Hz, 1H), 6.83 – 6.71 (m, 3H), 6.53 (d, *J* = 7.9 Hz, 1H), 6.37 (dd, *J* = 10.2, 3.3 Hz, 1H), 5.73 (dd, *J* = 10.2, 1.6 Hz, 1H), 4.56 (s, 1H), 3.90 (d, *J* = 13.5 Hz, 1H), 3.65 (d, *J* = 13.5 Hz, 1H), 3.27 (s, 3H), 2.39 (s, 3H), 2.35 (s, 3H). ^13^C NMR (101 MHz, CDCl_3_) δ 177.95, 143.56, 140.30, 138.58, 136.45, 135.31, 132.81, 129.20, 128.77, 128.14, 128.07, 127.38, 127.36, 126.72, 126.44, 124.91, 124.23, 123.72, 109.01, 57.97, 57.86, 54.11, 38.09, 26.58, 21.79. HRMS (EI) calculated for [C_27_H_26_N_2_O]^+^: 394.2045 found: 394.2041. Enantiomeric excess was determined by HPLC with a Chiralpak AD-H column (hexanes/2-propanol = 82/18, 1.0 mL/min, 210 nm); minor enantiomer *t_R_* = 5.6 min, major enantiomer *t_R_* = 7.3 min. [α]_D_^20^ = 139.2 (*c* = 0.3, CHCl_3_).

4.28 Synthesis of (3*R*,4'*S*)-4'-(benzyl(methyl)amino)-6-methoxy-1-methyl-4'*H*-spiro[indoline-3,1'-naphthalen]-2-one (**3ab**)

Prepared according to typical procedure from **1ab** (125.2 mg, 0.3 mmol), after a flash column chromatography (petroleum ether/ethyl acetate = 6/1) afforded the product **3ab** as a white solid (106.3 mg, 86% yield) with 92% *ee*. Mp: 144-146 ^o^C. ^1^H NMR (400 MHz, CDCl_3_) δ 7.77 (d, *J* = 7.9 Hz, 1H), 7.41 (d, *J* = 7.0 Hz, 2H), 7.32 (t, *J* = 7.6 Hz, 2H), 7.29 – 7.21 (m, 2H), 7.05 (td, *J* = 7.5, 1.4 Hz, 1H), 6.76 (d, *J* = 8.1 Hz, 1H), 6.58 – 6.46 (m, 3H), 6.36 (dd, *J* = 10.2, 3.3 Hz, 1H), 5.73 (dd, *J* = 10.2, 1.6 Hz, 1H), 4.55 (s, 1H), 3.90 (d, *J* = 13.5 Hz, 1H), 3.82 (s, 3H), 3.65 (d, *J* = 13.5 Hz, 1H), 3.26 (s, 3H), 2.35 (s, 3H). ^13^C NMR (101 MHz, CDCl_3_) δ 178.19, 160.39, 144.75, 140.32, 136.48, 135.50, 129.21, 128.78, 128.23, 128.15, 127.79, 127.38, 126.73, 126.45, 125.14, 124.83, 106.95, 96.09, 57.97, 57.88, 55.53, 53.80, 38.10, 26.65. HRMS (EI) calculated for [C_27_H_26_N_2_O_2_]^+^: 410.1994 found: 410.1992. Enantiomeric excess was determined by HPLC with a Chiralpak AD-H column (hexanes/2-propanol = 85/15, 1.0 mL/min, 210 nm); minor enantiomer *t_R_* = 7.0 min, major enantiomer *t_R_* = 11.2 min. [α]_D_^20^ = 140.7 (*c* = 0.3, CHCl_3_).

4.29 Synthesis of (3*R*,4'*S*)-4'-(benzyl(methyl)amino)-6-fluoro-1-methyl-4'*H*-spiro[indoline-3,1'-naphthalen]-2-one (**3ac**)

Prepared according to typical procedure from **1ac** (121.6 mg, 0.3 mmol), after a flash column chromatography (petroleum ether/ethyl acetate = 8/1) afforded the product **3ac** as a white solid (84.9 mg, 71% yield) with 92% *ee*. Mp: 146-148 ^o^C. ^1^H NMR (400 MHz, CDCl_3_) δ 7.78 (d, *J* = 7.8 Hz, 1H), 7.41 (d, *J* = 7.0 Hz, 2H), 7.35 – 7.21 (m, 4H), 7.06 (td, *J* = 7.5, 1.5 Hz, 1H), 6.83-6.75 (m, 1H), 6.70 – 6.62 (m, 2H), 6.51 (dd, *J* = 7.9, 1.3 Hz, 1H), 6.39 (dd, *J* = 10.2, 3.3 Hz, 1H), 5.71 (dd, *J* = 10.2, 1.7 Hz, 1H), 4.55 (s, 1H), 3.89 (d, *J* = 13.5 Hz, 1H), 3.65 (d, *J* = 13.5 Hz, 1H), 3.25 (s, 3H), 2.35 (s, 3H). ^13^C NMR (101 MHz, CDCl_3_) δ 177.95, 163.20 (d, *J* = 245.5 Hz), 145.04 (d, *J* = 11.4 Hz), 140.28, 136.58, 134.90, 131.01 (d, *J* = 3.0 Hz), 129.41, 128.84, 128.25, 127.68 (d, *J* = 2.1 Hz), 127.54, 126.86, 126.40, 125.76, 125.66, 125.47, 109.33 (d, *J* = 22.4 Hz), 96.99 (d, *J* = 27.6 Hz), 57.96, 53.91, 38.15, 26.84. ^19^F NMR (376 MHz, CDCl_3_) δ -111.63 (td, *J* = 9.1, 5.3 Hz). HRMS (ESI) calculated for C_26_H_24_FN_2_O: 399.1867 (M+H^+^), found: 399.1864. Enantiomeric excess was determined by HPLC with a Chiralpak AD-H column (hexanes/2-propanol = 82/18, 1.0 mL/min, 210 nm); minor enantiomer *t_R_* = 5.6 min, major enantiomer *t_R_* = 13.3 min. [α]_D_^20^ = 164.8 (*c* = 0.3, CHCl_3_).

4.30 Synthesis of (3*R*,4'*S*)-4'-(benzyl(methyl)amino)-6-chloro-1-methyl-4'*H*-spiro[indoline-3,1'-naphthalen]-2-one (**3ad**)

Prepared according to typical procedure from **1ad** (126.5 mg, 0.3 mmol), after a flash column chromatography (petroleum ether/ethyl acetate = 8/1) afforded the product **3ad** as a white solid (88.8 mg, 71% yield) with 93% *ee*. Mp: 189-193 ^o^C. ^1^H NMR (400 MHz, CDCl_3_) δ 7.78 (dd, *J* = 7.9, 1.4 Hz, 1H), 7.41 (d, *J* = 6.8 Hz, 2H), 7.35 – 7.21 (m, 4H), 7.07 (td, *J* = 7.6, 1.5 Hz, 1H), 7.00 – 6.91 (m, 2H), 6.77 (d, *J* = 7.9 Hz, 1H), 6.51 (dd, *J* = 7.9, 1.3 Hz, 1H), 6.40 (dd, *J* = 10.2, 3.3 Hz, 1H), 5.71 (dd, *J* = 10.2, 1.7 Hz, 1H), 4.55 (s, 1H), 3.89 (d, *J* = 13.5 Hz, 1H), 3.65 (d, *J* = 13.5 Hz, 1H), 3.28 (s, 3H), 2.35 (s, 3H). ^13^C NMR (101 MHz, CDCl_3_) δ 177.59, 144.75, 140.22, 136.53, 134.56, 134.23, 134.00, 129.41, 128.81, 128.23, 127.76, 127.55, 127.32, 126.85, 126.37, 125.67, 125.57, 123.12, 108.90, 57.94, 57.90, 53.99, 38.11, 26.80. HRMS (EI) calculated for [C_26_H_23_ClN_2_O]^+^: 414.1499 found: 414.1496. Enantiomeric excess was determined by HPLC with a Chiralpak AD-H column (hexanes/2-propanol = 85/15, 1.0 mL/min, 210 nm); minor enantiomer *t_R_* = 5.5 min, major enantiomer *t_R_* = 12.5 min. [α]_D_^20^ = 129.9 (*c* = 0.3, CHCl_3_).

4.31 Synthesis of (3*R*,4'*S*)-4'-(benzyl(methyl)amino)-6-bromo-1-methyl-4'*H*-spiro[indoline-3,1'-naphthalen]-2-one (**3ae**)

Prepared according to typical procedure from **1ae** (139.8 mg, 0.3 mmol), after a flash column chromatography (petroleum ether/ethyl acetate = 8/1) afforded the product **3ae** as a pale yellow solid (64.9 mg, 47% yield) with 94% *ee*. Mp: 182-184 ^o^C. ^1^H NMR (400 MHz, CDCl_3_) δ 7.78 (d, *J* = 7.9 Hz, 1H), 7.40 (d, *J* = 6.8 Hz, 2H), 7.33 – 7.21 (m, 4H), 7.14 – 7.01 (m, 3H), 6.71 (d, *J* = 7.9 Hz, 1H), 6.51 (dd, *J* = 7.9, 1.3 Hz, 1H), 6.39 (dd, *J* = 10.2, 3.3 Hz, 1H), 5.70 (dd, *J* = 10.2, 1.7 Hz, 1H), 4.55 (s, 1H), 3.89 (d, *J* = 13.4 Hz, 1H), 3.65 (d, *J* = 13.5 Hz, 1H), 3.26 (s, 3H), 2.34 (s, 3H). ^13^C NMR (101 MHz, CDCl_3_) δ 177.44, 144.93, 140.23, 136.54, 134.57, 134.49, 129.44, 128.83, 128.26, 127.79, 127.58, 127.24, 126.87, 126.40, 126.08, 125.96, 125.72, 122.00, 111.69, 57.96, 57.92, 54.06, 38.14, 26.82. HRMS (ESI) calculated for C_26_H_24_BrN_2_O: 459.1067 (M+H^+^), found: 459.1062. Enantiomeric excess was determined by HPLC with a Chiralpak AD-H column (hexanes/2-propanol = 85/15, 1.0 mL/min, 210 nm); minor enantiomer *t_R_* = 5.7 min, major enantiomer *t_R_* = 12.0 min. [α]_D_^20^ = 111.2 (*c* = 0.3, CHCl_3_).

4.32 Synthesis of methyl (3*R*,4'*S*)-4'-(benzyl(methyl)amino)-1-methyl-2-oxo-4'*H*-spiro[indoline-3,1'-naphthalene]-6-carboxylate (**3af**)

Prepared according to typical procedure from **1af** (133.6 mg, 0.3 mmol), after a flash column chromatography (petroleum ether/ethyl acetate = 8/1) afforded the product **3af** as a pale yellow solid (78.7 mg, 60% yield) with 94% *ee*. Mp: 78-80 ^o^C. ^1^H NMR (400 MHz, CDCl_3_) δ 7.80 (d, *J* = 7.8 Hz, 1H), 7.71 (dd, *J* = 7.7, 1.5 Hz, 1H), 7.59 (d, *J* = 1.4 Hz, 1H), 7.41 (d, *J* = 7.0 Hz, 2H), 7.35 – 7.21 (m, 4H), 7.06 (td, *J* = 7.6, 1.5 Hz, 1H), 6.92 (d, *J* = 7.7 Hz, 1H), 6.49 (dd, *J* = 7.8, 1.3 Hz, 1H), 6.43 (dd, *J* = 10.2, 3.3 Hz, 1H), 5.73 (dd, *J* = 10.2, 1.7 Hz, 1H), 4.58 (s, 1H), 3.93 (s, 3H), 3.90 (d, *J* = 13.4 Hz, 1H), 3.67 (d, *J* = 13.4 Hz, 1H), 3.34 (s, 3H), 2.36 (s, 3H). ^13^C NMR (101 MHz, CDCl_3_) δ 177.31, 166.61, 143.83, 140.56, 140.15, 136.46, 134.24, 130.56, 129.42, 128.77, 128.19, 127.80, 127.55, 126.91, 126.81, 126.32, 125.90, 125.12, 124.43, 108.87, 57.91, 57.85, 54.39, 52.31, 38.06, 26.85. HRMS (EI) calculated for [C_28_H_26_N_2_O_3_]^+^: 438.1943 found: 438.1939. Enantiomeric excess was determined by HPLC with a Chiralpak AD-H column (hexanes/2-propanol = 85/15, 1.0 mL/min, 210 nm); minor enantiomer *t_R_* = 10.8 min, major enantiomer *t_R_* = 14.0 min. [α]_D_^20^ = 148.1 (*c* = 0.3, CHCl_3_).

4.33 Synthesis of methyl (3*R*,4'*S*)-4'-(benzyl(met hyl)amino)-1-methyl-2-oxo-4'*H*-spiro[indoline-3,1'-naphthalene]-6-carboxylate (**3ag**)

Prepared according to typical procedure from **1ag** (126.5 mg, 0.3 mmol), after a flash column chromatography (petroleum ether/ethyl acetate = 8/1) afforded the product **3ag** as a pale yellow solid (88.1 mg, 74% yield) with 93% *ee*. Mp: 111-114 ^o^C. ^1^H NMR (400 MHz, CDCl_3_) δ 7.79 (d, *J* = 7.9 Hz, 1H), 7.41 (d, *J* = 6.8 Hz, 2H), 7.34 – 7.19 (m, 4H), 7.12 – 7.00 (m, 2H), 6.80 (d, *J* = 7.9 Hz, 1H), 6.68 (d, *J* = 1.8 Hz, 1H), 6.54 (dd, *J* = 7.9, 1.4 Hz, 1H), 6.38 (dd, *J* = 10.2, 3.3 Hz, 1H), 5.74 (dd, *J* = 10.2, 1.7 Hz, 1H), 4.58 (s, 1H), 3.90 (d, *J* = 13.5 Hz, 1H), 3.66 (d, *J* = 13.5 Hz, 1H), 3.26 (s, 3H), 2.36 (s, 3H), 2.21 (s, 3H). ^13^C NMR (101 MHz, CDCl_3_) δ 177.59, 141.09, 140.27, 136.45, 135.64, 135.22, 132.80, 129.19, 128.76, 128.63, 128.13, 127.99, 127.40, 127.38, 126.71, 126.50, 125.27, 124.93, 107.82, 57.93, 57.86, 54.38, 38.07, 26.63, 20.94. HRMS (EI) calculated for [C_27_H_26_N_2_O]^+^: 394.2045 found: 394.2043. Enantiomeric excess was determined by HPLC with a Chiralpak AD-H column (hexanes/2-propanol = 85/15, 1.0 mL/min, 210 nm); minor enantiomer *t_R_* = 6.2 min, major enantiomer *t_R_* = 8.7 min. [α]_D_^20^ = 159.8 (*c* = 0.3, CHCl_3_).

4.34 Synthesis of methyl (3*R*,4'*S*)-4'-(benzyl(methyl)amino)-5-fluoro-1-methyl-4'*H*-spiro[indoline-3,1'-naphthalen]-2-one (**3ah**)

Prepared according to typical procedure from **1ah** (121.5 mg, 0.3 mmol), after a flash column chromatography (petroleum ether/ethyl acetate = 8/1) afforded the product **3ah** as a white solid (69.9 mg, 47% yield) with 93% *ee*. Mp: 129-132 ^o^C. ^1^H NMR (400 MHz, CDCl_3_) δ 7.84 – 7.76 (m, 1H), 7.41 (d, *J* = 7.1 Hz, 2H), 7.35 – 7.21 (m, 4H), 7.10 – 7.05 (m, 1H), 6.99 (td, *J* = 8.8, 2.6 Hz, 1H), 6.84 (dd, *J* = 8.5, 4.1 Hz, 1H), 6.62 (dd, *J* = 7.7, 2.6 Hz, 1H), 6.52 (dd, *J* = 7.8, 1.3 Hz, 1H), 6.41 (dd, *J* = 10.2, 3.3 Hz, 1H), 5.73 (dd, *J* = 10.2, 1.7 Hz, 1H), 4.56 (s, 1H), 3.89 (d, *J* = 13.4 Hz, 1H), 3.66 (d, *J* = 13.5 Hz, 1H), 3.28 (s, 3H), 2.35 (s, 3H). ^13^C NMR (101 MHz, CDCl_3_) δ 177.39, 159.63 (d, *J* = 241.4 Hz), 140.19, 139.43 (d, *J* = 1.9 Hz), 137.11 (d, *J* = 8.2 Hz), 136.47, 134.42, 129.40, 128.77, 128.19, 127.75, 127.52, 127.21, 126.80, 126.34, 125.73, 114.72 (d, *J* = 23.7 Hz), 112.64 (d, *J* = 24.8 Hz), 108.60 (d, *J* = 7.9 Hz), 57.89, 54.64, 38.08, 26.80. ^19^F NMR (376 MHz, CDCl_3_) δ -119.70 (td, *J* = 8.4, 4.0 Hz). HRMS (EI) calculated for [C_26_H_23_FN_2_O]^+^: 398.1794 found: 398.1790. Enantiomeric excess was determined by HPLC with a Chiralpak AD-H column (hexanes/2-propanol = 82/18, 1.0 mL/min, 210 nm); minor enantiomer *t_R_* = 5.0 min, major enantiomer *t_R_* = 6.8 min. [α]_D_^20^ = 124.3 (*c* = 0.3, CHCl_3_).

4.35 Synthesis of (3*R*,4'*S*)-4'-(benzyl(methyl)amino)-5-bromo-1-methyl-4'*H*-spiro[indoline-3,1'-naphthalen]-2-one (**3ai**)

Prepared according to typical procedure from **1ai** (139.8 mg, 0.3 mmol), after a flash column chromatography (petroleum ether/ethyl acetate = 8/1) afforded the product **3ai** as a pale yellow solid (67.5 mg, 49% yield) with 87% *ee*. Mp: 97-100 ^o^C. ^1^H NMR (400 MHz, CDCl_3_) δ 7.80 (d, *J* = 7.8 Hz, 1H), 7.45-7.38 (m, 3H), 7.34 – 7.22 (m, 4H), 7.08 (td, *J* = 7.6, 1.5 Hz, 1H), 6.97 (d, *J* = 2.0 Hz, 1H), 6.80 (d, *J* = 8.3 Hz, 1H), 6.51 (dd, *J* = 7.9, 1.4 Hz, 1H), 6.41 (dd, *J* = 10.2, 3.3 Hz, 1H), 5.72 (dd, *J* = 10.2, 1.7 Hz, 1H), 4.56 (s, 1H), 3.88 (d, *J* = 13.5 Hz, 1H), 3.65 (d, *J* = 13.5 Hz, 1H), 3.27 (s, 3H), 2.35 (s, 3H). ^13^C NMR (101 MHz, CDCl_3_) δ 177.14, 142.61, 140.20, 137.51, 136.53, 134.31, 131.34, 129.47, 128.81, 128.25, 127.85, 127.62, 127.10, 126.86, 126.41, 125.89, 115.85, 109.65, 57.93, 57.92, 54.37, 38.11, 26.80. HRMS (ESI) calculated for C_26_H_24_BrN_2_O: 459.1067 (M+H^+^), found: 459.1075. Enantiomeric excess was determined by HPLC with a Chiralpak AD-H column (hexanes/2-propanol = 85/15, 1.0 mL/min, 210 nm); minor enantiomer *t_R_* = 5.5 min, major enantiomer *t_R_* = 7.8 min. [α]_D_^20^ = 164.5 (*c* = 0.3, CHCl_3_).

4.36 Synthesis of (3*R*,4'*S*)-1-benzyl-4'-morpholino-4'*H*-spiro[indoline-3,1'-naphthalen]-2-one (**3aj**)

Prepared according to typical procedure from **1aj** (139 mg, 0.3 mmol), after a flash column chromatography (petroleum ether/ethyl acetate = 5/1) afforded the product **3aj** as a white solid (117.8 mg, 93% yield) with 89% *ee*. Mp: 120-122 ^o^C. ^1^H NMR (400 MHz, CDCl_3_) δ 7.74 (dd, *J* = 8.0, 1.3 Hz, 1H), 7.42 – 7.30 (m, 4H), 7.29 – 7.22 (m, 2H), 7.17 (td, *J* = 7.7, 1.4 Hz, 1H), 7.06 (td, *J* = 7.6, 1.4 Hz, 1H), 6.94 (t, *J* = 7.5 Hz, 1H), 6.91 – 6.79 (m, 2H), 6.55 (dd, *J* = 7.9, 1.3 Hz, 1H), 6.34 (dd, *J* = 10.2, 3.5 Hz, 1H), 5.78 (dd, *J* = 10.2, 1.6 Hz, 1H), 5.04 (d, *J* = 15.4 Hz, 1H), 4.87 (d, *J* = 15.5 Hz, 1H), 4.59 – 4.43 (m, 1H), 3.73 (ddt, *J* = 27.8, 10.8, 4.5 Hz, 4H), 2.77 (t, *J* = 4.6 Hz, 4H). ^13^C NMR (101 MHz, CDCl_3_) δ 177.53, 142.52, 135.89, 135.44, 135.39, 135.26, 129.33, 128.78, 128.32, 127.90, 127.70, 127.49, 127.47, 127.40, 126.47, 125.43, 124.58, 123.23, 109.15, 67.75, 59.94, 54.15, 49.34, 44.10. HRMS (ESI) calculated for C_28_H_27_N_2_O_2_: 423.2067 (M+H^+^), found: 423.2067. Enantiomeric excess was determined by HPLC with a Chiralpak AD-H column (hexanes/2-propanol = 80/20, 1.0 mL/min, 210 nm); minor enantiomer *t_R_* = 13.7 min, major enantiomer *t_R_* = 19.3 min. [α]_D_^20^ = 111.8 (*c* = 0.2, CHCl_3_).

4.37 Synthesis of (3*R*,4'*S*)-1-methyl-4'-(4-phenylpiperazin-1-yl)-4'*H*-spiro[indoline-3,1'-naphthalen]-2-one (**3ak**)

Prepared according to typical procedure from **2ak**(97.3 mg, 0.6 mmol), after a flash column chromatography (petroleum ether/ethyl acetate = 6/1) afforded the product **3ak** as a white solid (78.0 mg, 62% yield) with 86% *ee*. Mp: 85-87 ^o^C. ^1^H NMR (400 MHz, CDCl_3_) δ 7.74 (d, *J* = 7.8 Hz, 1H), 7.30 (td, *J* = 7.7, 1.3 Hz, 1H), 7.27 – 7.19 (m, 3H), 7.06 (td, *J* = 7.6, 1.5 Hz, 1H), 7.00 (td, *J* = 7.5, 1.0 Hz, 1H), 6.96 – 6.87 (m, 4H), 6.81 (t, *J* = 7.3 Hz, 1H), 6.54 (dd, *J* = 7.9, 1.3 Hz, 1H), 6.34 (dd, *J* = 10.2, 3.4 Hz, 1H), 5.72 (dd, *J* = 10.2, 1.6 Hz, 1H), 4.57 (s, 1H), 3.30 (s, 3H), 3.28-3.22 (m, 2H), 3.22-3.14 (m, 2H), 2.96-2.85 (m, 4H). ^13^C NMR (101 MHz, CDCl_3_) δ 177.55, 151.60, 143.49, 135.56, 135.53, 135.17, 129.29, 128.97, 128.44, 127.80, 127.47, 127.44, 126.46, 125.58, 124.53, 123.25, 119.23, 115.97, 108.13, 59.73, 54.27, 49.90, 48.83, 26.64. HRMS (EI) calculated for [C_28_H_27_N_3_O]^+^: 421.2154 found: 421.2150. Enantiomeric excess was determined by HPLC with a Chiralpak AD-H column (hexanes/2-propanol = 75/25, 1.0 mL/min, 210 nm); minor enantiomer *t_R_* = 9.1 min, major enantiomer *t_R_* = 19.7 min. [α]_D_^20^ = 77.0 (*c* = 0.3, CHCl_3_).

4.38 Synthesis of (3*R*,4'*S*)-4'-(3,4-dihydroisoquinolin-2(1*H*)-yl)-1-methyl-4'*H*-spiro[indoline-3,1'-naphthalen]-2-one (**3al**)

Prepared according to typical procedure from **2al** (133.2 mg, 0.6 mmol), after a flash column chromatography (petroleum ether/ethyl acetate = 6/1) afforded the product **3al** as a yellow solid (76.3 mg, 65% yield) with 82% *ee*. Mp: 110-113 ^o^C. ^1^H NMR (400 MHz, CDCl_3_) δ 7.76 (dd, *J* = 7.9, 1.4 Hz, 1H), 7.31 (td, *J* = 7.7, 1.3 Hz, 1H), 7.21 (td, *J* = 7.5, 1.3 Hz, 1H), 7.15 – 7.04 (m, 5H), 7.01 (td, *J* = 7.5, 1.0 Hz, 1H), 6.97 – 6.88 (m, 2H), 6.57 (dd, *J* = 7.9, 1.3 Hz, 1H), 6.39 (dd, *J* = 10.2, 3.4 Hz, 1H), 5.76 (dd, *J* = 10.2, 1.7 Hz, 1H), 4.72 (s, 1H), 4.01 (d, *J* = 15.0 Hz, 1H), 3.90 (d, *J* = 15.0 Hz, 1H), 3.33 (s, 3H), 3.13-3.03 (m, 1H), 3.01-2.90 (m, 2H), 2.89 – 2.79 (m, 1H). ^13^C NMR (101 MHz, CDCl_3_) δ 177.78, 143.55, 136.04, 135.74, 135.67, 135.12, 134.98, 129.40, 128.82, 128.50, 128.05, 127.58, 126.86, 126.49, 125.77, 125.49, 125.37, 124.62, 123.33, 108.21, 59.67, 54.42, 51.61, 46.84, 30.46, 26.73. HRMS (ESI) calculated for [M+H^+^]: 393.1961 found: 393.1965. Enantiomeric excess was determined by HPLC with a Chiralpak AD-H column (hexanes/2-propanol = 75/25, 1.0 mL/min, 210 nm); minor enantiomer *t_R_* = 5.9 min, major enantiomer *t_R_* = 13.6 min. [α]_D_^20^ = 64.9 (*c* = 0.3, CHCl_3_).

4.39 Synthesis of (3*R*,4'*S*)-1-methyl-4'-((4-methyl-2-oxo-2*H*-chromen-7-yl)amino)-4'*H*-spiro[indoline-3,1'-naphthalen]-2-one (**5a**)

Prepared according to typical procedure from **4a** (70.1 mg, 0.4 mmol), after a flash column chromatography (petroleum ether/ethyl acetate = 4/1) afforded the product **5a** as a yellow solid (76.7 mg, 88% yield) with 95% *ee*. Mp: 135-138 ^o^C. ^1^H NMR (400 MHz, CDCl_3_) δ 7.50 (dd, *J* = 7.8, 1.4 Hz, 1H), 7.44 – 7.39 (m, 1H), 7.35 (d, *J* = 8.6 Hz, 1H), 7.28 – 7.21 (m, 1H), 7.14 (td, *J* = 7.5, 1.0 Hz, 1H), 7.09 (td, *J* = 7.6, 1.4 Hz, 2H), 7.00 (dd, *J* = 7.9, 0.8 Hz, 1H), 6.82 (d, *J* = 2.4 Hz, 1H), 6.68 (dd, *J* = 8.6, 2.4 Hz, 1H), 6.62 – 6.50 (m, 2H), 5.98 (q, *J* = 1.1 Hz, 1H), 5.82 (dd, *J* = 14.6, 9.9 Hz, 2H), 5.41 (dd, *J* = 10.2, 5.1 Hz, 1H), 3.27 (s, 3H), 2.33 (d, *J* = 1.2 Hz, 3H). ^13^C NMR (101 MHz, CDCl_3_) δ 176.58, 161.94, 156.01, 152.90, 150.76, 143.96, 136.50, 136.04, 133.48, 130.04, 129.02, 128.65, 128.16, 127.72, 126.39, 125.56, 124.80, 123.74, 111.77, 110.63, 109.21, 108.51, 98.35, 55.54, 49.95, 26.97, 18.44. HRMS (ESI) calculated for C_28_H_22_N_2_O_3_Na: 457.1523 (M+Na^+^), found: 457.1524. Enantiomeric excess was determined by HPLC with a Chiralpak OD-H column (hexanes/2-propanol = 80/20, 1.0 mL/min, 210 nm); minor enantiomer *t_R_* = 27.1 min, major enantiomer *t_R_* = 34.1 min. [α]_D_^20^ = -39.2 (*c* = 0.2, CHCl_3_).

4.40 Synthesis of (3*S*,4'*R*)-1-methyl-4'-((1*S*,5*R*)-8-oxo-1,5,6,8-tetrahydro-2*H*-1,5-methanopyrido[1,2-*a*][1,5]diazocin-3(4*H*)-yl)-4'*H*-spiro[indoline-3,1'-naphthalen]-2-one (**5b**)

Prepared according to typical procedure with *ent*-**M4** as chiral ligand from **4b** (76.1 mg, 0.4 mmol), after a flash column chromatography (petroleum ether/ethyl acetate/acetone = 1/3/1) afforded the product **5b** as a pale yellow solid (80.7 mg, 90% yield) with 15:1 *dr*. Mp: 121-123 ^o^C. ^1^H NMR (400 MHz, CDCl_3_) δ 7.34 (dd, *J* = 9.0, 6.8 Hz, 1H), 7.31 – 7.26 (m, 1H), 7.23 – 7.17 (m, 1H), 7.08 (dd, *J* = 8.0, 1.5 Hz, 1H), 7.05 – 6.91 (m, 3H), 6.83 (dd, *J* = 7.5, 1.3 Hz, 1H), 6.50 (ddd, *J* = 12.4, 8.5, 1.3 Hz, 2H), 6.17 (dd, *J* = 10.2, 3.4 Hz, 1H), 6.08 (dd, *J* = 6.9, 1.5 Hz, 1H), 5.67 (dd, *J* = 10.1, 1.6 Hz, 1H), 4.27 (dd, *J* = 3.4, 1.6 Hz, 1H), 3.79 – 3.72 (m, 2H), 3.29 (s, 3H), 3.20 (dq, *J* = 9.2, 2.0 Hz, 1H), 3.13 – 3.02 (m, 2H), 2.84 (ddt, *J* = 11.7, 3.5, 1.7 Hz, 1H), 2.57 (dd, *J* = 11.6, 2.0 Hz, 1H), 2.35 (tt, *J* = 4.8, 2.8 Hz, 1H), 1.89 (dtd, *J* = 14.2, 3.3, 1.6 Hz, 1H), 1.79 (dt, *J* = 12.8, 3.1 Hz, 1H). ^13^C NMR (101 MHz, CDCl_3_) δ 177.41, 163.40, 151.89, 143.33, 138.48, 135.20, 135.10, 134.66, 128.44, 128.37, 127.95, 127.85, 127.55, 126.18, 124.89, 124.38, 123.15, 116.30, 108.07, 104.29, 59.34, 59.06, 54.04, 53.14, 49.42, 36.15, 27.67, 26.53, 26.06. HRMS (ESI) calculated for C_29_H_28_N_2_O_2_: 450.2176 (M+H^+^), found: 450.2178. [α]_D_^20^ = -240.8 (*c* = 0.2, CHCl_3_).

4.41 Synthesis of (3*S*,4'*R*)-1-methyl-4'-((1*S*,5*R*)-8-oxo-1,5,6,8-tetrahydro-2*H*-1,5-methanopyrido[1,2-*a*][1,5]diazocin-3(4*H*)-yl)-4'*H*-spiro[indoline-3,1'-naphthalen]-2-one (**5c**)

Prepared according to typical procedure from **4c** (118.2 mg, 0.4 mmol), after a flash column chromatography (petroleum ether/ethyl acetate = 5/1) afforded the product **5c** as a white solid (91.5 mg, 82% yield) with 94% *ee*. Mp: 141-143 ^o^C. ^1^H NMR (400 MHz, CDCl_3_) δ 7.80 – 7.71 (m, 1H), 7.50 – 7.42 (m, 1H), 7.37 (dt, *J* = 7.7, 1.8 Hz, 1H), 7.33 – 7.19 (m, 5H), 7.19 – 7.11 (m, 1H), 7.11 – 7.03 (m, 2H), 7.02 – 6.96 (m, 1H), 6.94 – 6.80 (m, 3H), 6.52 (ddd, *J* = 7.9, 4.8, 1.3 Hz, 1H), 6.34 (ddd, *J* = 24.5, 10.2, 3.4 Hz, 1H), 5.71 (ddd, *J* = 11.6, 10.1, 1.6 Hz, 1H), 4.57 (dt, *J* = 11.8, 2.6 Hz, 1H), 3.43 (d, *J* = 9.7 Hz, 4H), 3.27 (d, *J* = 1.7 Hz, 3H), 2.79 (s, 4H). ^13^C NMR (101 MHz, CDCl_3_) δ 177.58, 177.47, 161.29, 160.43, 149.09, 148.93, 143.41, 139.70 (d, *J* = 8.4 Hz), 135.40, 135.05 (d, *J* = 13.8 Hz), 134.08 (d, *J* = 5.1 Hz), 132.11 – 131.90 (m), 130.50 (d, *J* = 3.0 Hz), 129.31 – 129.11 (m), 128.93, 128.45, 128.26, 128.03, 127.92, 127.82, 127.46 (d, *J* = 3.8 Hz), 126.41 (d, *J* = 10.3 Hz), 125.60, 125.45, 125.26 (d, *J* = 4.1 Hz), 124.51 (d, *J* = 2.5 Hz), 123.27 (d, *J* = 3.4 Hz), 122.48 (d, *J* = 20.0 Hz), 108.15, 108.13, 59.82, 59.73, 54.21, 48.85, 26.60 (d, *J* = 1.4 Hz). HRMS (ESI) calculated for C_35_H_31_N_4_OS: 555.2213 (M+H^+^), found: 555.2214. Enantiomeric excess was determined by HPLC with a Chiralpak IA column (hexanes/2-propanol = 90/10, 1.0 mL/min, 254 nm); minor enantiomer *t_R_* = 15.0 min, major enantiomer *t_R_* = 24.8 min. [α]_D_^20^ = 90.9 (*c* = 0.2, CHCl_3_).

4.42 Synthesis of (3*R*,4'*S*)-4'-((3*S*,4*R*)-3-((benzo[*d*][1,3]dioxol-5-yloxy)methyl)-4-(4-fluorophenyl)piperidin-1-yl)-1-methyl-4'*H*-spiro[indoline-3,1'-naphthalen]-2-one (**5d**)

Prepared according to typical procedure from **4d** (131.8 mg, 0.4 mmol), after a flash column chromatography (petroleum ether/ethyl acetate = 5/1) afforded the product **5d** as a white solid (94.4 mg, 78% yield) with > 20:1 *dr*. Mp: 176-177 ^o^C. ^1^H NMR (400 MHz, CDCl_3_) δ 7.73 (d, *J* = 7.8 Hz, 1H), 7.32 – 7.22 (m, 2H), 7.21 – 7.13 (m, 2H), 7.08 (td, *J* = 7.6, 1.4 Hz, 1H), 7.02 – 6.90 (m, 4H), 6.87 (dd, *J* = 7.4, 1.3 Hz, 1H), 6.61 – 6.54 (m, 2H), 6.36 (dd, *J* = 10.2, 3.4 Hz, 1H), 6.31 (d, *J* = 2.5 Hz, 1H), 6.09 (dd, *J* = 8.5, 2.5 Hz, 1H), 5.83 (s, 2H), 5.72 (dd, *J* = 10.1, 1.8 Hz, 1H), 4.60 (s, 1H), 3.55 (dd, *J* = 9.5, 3.2 Hz, 1H), 3.43 (dd, *J* = 9.4, 6.4 Hz, 1H), 3.29 (s, 4H), 3.22 – 3.12 (m, 1H), 2.61 (td, *J* = 10.9, 3.6 Hz, 1H), 2.49 (td, *J* = 11.3, 4.2 Hz, 2H), 2.08 (s, 1H), 1.92 (m, 2H). ^13^C NMR (101 MHz, CDCl_3_) δ 177.56, 161.35 (d, *J* = 243.8 Hz), 154.45, 147.87, 143.41, 141.38, 140.23, 135.66, 135.29, 128.86 (d, *J* = 7.9 Hz), 128.37, 127.45, 127.26, 126.68, 124.46, 123.18, 115.12 (d, *J* = 21.0 Hz), 108.09, 107.63, 106.19, 100.87, 98.52, 70.08, 60.22, 54.31, 52.28, 50.90, 44.54, 42.83, 35.26, 26.64.^19^F NMR (376 MHz, CDCl_3_) δ -116.45, -116.81, -117.00. HRMS (ESI) calculated for C_37_H_34_FN_2_O_4_: 589.2497 (M+H^+^), found: 589.2497. [α]_D_^20^ = 20.9 (*c* = 0.2, CHCl_3_).

5. **Gram-scale synthesis.**

Under nitrogen atmosphere, to an oven-dried 100 mL Schlenk tube equipped with a magnetic stir was added [*η*-PhC_3_H_4_PdCl]_2_ (64.0 mg, 0.125 mmol, 2.5 mol%), **M4** (288.4 mg, 0.35 mmol, 7 mol%) and DCM (12.5 mL). The solution was stirred for 40 min at room temperature. Then **1a** (5.0 mmol, 1.0 equiv), **2e** (10.0 mmol, 2.0 equiv), Ag_3_PO_4_ (2.09 g, 5.0 mmol, 1.0 equiv), Na_2_HPO_4_·12H_2_O (3.58 g, 10.0 mmol, 2.0 equiv) and DCM (12.5 ml) were added to the solution. The mixture was then stirred at 80 ^o^C for about 48 h. After the reaction was completed (monitored by TLC), the reaction mixture was concentrated to dryness and the residue was purified by column chromatography (petroleum ether/ethyl acetate = 5/1) to afford desired product **3e** as a white solid (1.195 g, 69% yield) with 95% *ee*. Enantiomeric excess was determined by HPLC with a Chiralpak AD-H column (hexanes/2-propanol = 85/15, 1.0 mL/min, 210 nm); minor enantiomer *t_R_* = 12.3 min, major enantiomer *t_R_* = 14.7 min.

Under nitrogen atmosphere, to an oven-dried 100 mL Schlenk tube equipped with a magnetic stir was added [*η*-PhC_3_H_4_PdCl]_2_ (64.0 mg, 0.125 mmol, 2.5 mol%), **M4** (288.4 mg, 0.35 mmol, 7 mol%) and DCM (12.5 mL). The solution was stirred for 40 min at room temperature. Then **1a** (5.0 mmol, 1.0 equiv), **2g** (10.0 mmol, 2.0 equiv), Ag_3_PO_4_ (2.09 g, 5.0 mmol, 1.0 equiv), Na_2_HPO_4_·12H_2_O (3.58 g, 10.0 mmol, 2.0 equiv) and DCM (12.5 ml) were added to the solution. The mixture was then stirred at 80 ^o^C for about 48 h. After the reaction was completed (monitored by TLC), the reaction mixture was concentrated to dryness and the residue was purified by column chromatography (petroleum ether/ethyl acetate = 8/1) to afford desired product **3g** as a white solid (1.158 g, 66% yield) with 95% *ee*. Enantiomeric excess was determined by HPLC with a Chiralpak AD-H column (hexanes/2-propanol = 75/25, 1.0 mL/min, 210 nm); minor enantiomer *t_R_* = 6.6 min, major enantiomer *t_R_* = 10.6 min.

6. **Synthesis applications.**

6.1 Synthesis of (*R*)-1-methyl-2'*H*-spiro[indoline-3,1'-naphthalen]-2-one (**6**) and (*R*)-1-methyl-4'*H*-spiro[indoline-3,1'-naphthalen]-2-one (**7**).

A flame-dried flask was cooled down to room temperature under argon. To this tube were added **3e** (103.9 mg, 0.3 mmol), [RhCl(cod)]_2_ (3 mg, 0.006 mmol), Zn (39.2 mg, 0.6 mmol) H_2_O (0.12 mL), and 1,4-dioxane (0.6 mL). Then the reaction mixture was subjected to hydrogen (1 atm). The mixture was stirred at 120 ^o^C for 20 h. After completion, the mixture was filtered and the filtrate was concentrated under reduced pressure.^3^ The crude product was purified by silica gel column chromatography (petroleum ether/ethyl acetate = 15/1) to afford **6** as a colorless solid (35.3 mg, 45% yield with 94% *ee*) and **7** as a colorless solid (25.2 mg, 32% yield with 94% ee).

Compound **6**: Mp: 146-147 ^o^C. ^1^H NMR (400 MHz, CDCl_3_) δ 7.31 (dd, *J* = 7.4, 1.2 Hz, 1H), 7.24 (ddd, *J* = 9.0, 7.1, 1.2 Hz, 1H), 7.21 – 7.13 (m, 2H), 7.04 (td, *J* = 7.1, 2.3 Hz, 1H), 6.95 – 6.89 (m, 1H), 6.88 (d, *J* = 7.7 Hz, 1H), 6.71 (d, *J* = 7.7 Hz, 1H), 6.67 (dd, *J* = 9.7, 2.8 Hz, 1H), 6.03 (ddd, *J* = 9.2, 5.6, 3.1 Hz, 1H), 3.32 (s, 3H), 3.06 (dt, *J* = 17.4, 3.0 Hz, 1H), 2.54 – 2.41 (m, 1H). ^13^C NMR (101 MHz, CDCl_3_) δ 179.83, 141.54, 134.62, 134.36, 133.24, 128.35, 128.13, 127.85, 127.18, 125.62, 124.69, 123.52, 122.69, 108.26, 51.80, 33.74, 26.48. HRMS (ESI) calculated for C_18_H_16_NO: 262.1226 (M+H^+^), found: 262.1229. Enantiomeric excess was determined by HPLC with a Chiralpak ADH column (hexanes/2-propanol = 95/5, 1.0 mL/min, 254 nm); minor enantiomer *t_R_* = 11.0 min, major enantiomer *t_R_* = 14.2 min. [α]_D_^20^ = -151.9 (*c* = 0.2, CHCl_3_).

Compound **7**: Mp: 104-106 ^o^C. ^1^H NMR (400 MHz, CDCl_3_) δ 7.32 (ddd, *J* = 7.8, 6.9, 2.0 Hz, 1H), 7.27 – 7.22 (m, 1H), 7.18 (td, *J* = 7.4, 1.4 Hz, 1H), 7.07 – 6.98 (m, 3H), 6.92 (d, *J* = 7.8 Hz, 1H), 6.54 (dd, *J* = 7.9, 1.3 Hz, 1H), 6.32 (ddd, *J* = 9.9, 4.2, 3.1 Hz, 1H), 5.57 (ddd, *J* = 9.9, 2.6, 1.7 Hz, 1H), 3.79 (dt, *J* = 21.8, 3.0 Hz, 1H), 3.58 (ddd, *J* = 21.9, 4.3, 1.7 Hz, 1H), 3.26 (s, 3H). ^13^C NMR (101 MHz, CDCl_3_) δ 178.22, 143.65, 135.75, 134.28, 134.14, 128.76, 128.37, 128.02, 127.29, 126.76, 126.60, 125.40, 124.72, 123.22, 108.05, 54.72, 30.02, 26.61. HRMS (ESI) calculated for C_18_H_16_NO: 262.1226 (M+H^+^), found: 262.1228. Enantiomeric excess was determined by HPLC with a Chiralpak ADH column (hexanes/2-propanol = 95/5, 1.0 mL/min, 210 nm); minor enantiomer *t_R_* = 12.3 min, major enantiomer *t_R_* = 16.4 min. [α]_D_^20^ = 37.1 (*c* = 0.2, CHCl_3_).

6.2 Synthesis of (*R*)-1-methyl-3',4'-dihydro-2'*H*-spiro[indoline-3,1'-naphthalen]-2-one (**8**).

A flame-dried Schlenk tube was cooled down to room temperature under argon. To this tube were added **3e** (103.9 mg, 0.3 mmol), THF (6.0 mL) and Pd/C (5%, 45 mg). Then the reaction mixture was subjected to hydrogen (1 atm). The mixture was stirred at room temperature for 24 h. After completion, the mixture was filtered and the filtrate was concentrated under reduced pressure. The crude product was purified by silica gel column chromatography (petroleum ether/ethyl acetate = 8/1) to afford **8** as a pale yellow solid (66.5 mg, 84% yield with 93% *ee*).^4^ Mp: 101-104 ^o^C. ^1^H NMR (400 MHz, CDCl_3_) δ 7.28 (tt, *J* = 7.6, 1.3 Hz, 1H), 7.15 (d, *J* = 7.7 Hz, 1H), 7.13 – 7.07 (m, 1H), 7.07 – 7.02 (m, 1H), 7.02 – 6.98 (m, 1H), 6.98 – 6.89 (m, 2H), 6.47 (d, *J* = 7.8 Hz, 1H), 3.27 (s, 3H), 3.11-2.86 (m, 2H), 2.43 – 2.29 (m, 1H), 2.29 – 2.12 (m, 1H), 2.12 – 1.88 (m, 2H). ^13^C NMR (101 MHz, CDCl_3_) δ 180.63, 143.10, 137.84, 137.41, 135.13, 129.69, 128.00, 127.91, 127.12, 126.37, 124.03, 122.90, 108.10, 52.28, 34.06, 29.27, 26.54, 18.80. HRMS (EI) calculated for [C_18_H_17_NO]^+^: 263.1310 found: 263.1308. Enantiomeric excess was determined by HPLC with a Chiralpak AD-H column (hexanes/2-propanol = 80/20, 1.0 mL/min, 210 nm); minor enantiomer *t_R_* = 5.2 min, major enantiomer *t_R_* = 7.0 min. [α]_D_^20^ = -3.71 (*c* = 0.3, CHCl_3_).

6.3 Synthesis of (*R*)-1-methyl-3',4'-dihydro-2'*H*-spiro[indoline-3,1'-naphthalene] (**9**).

A flame-dried flask was cooled down to room temperature under argon. In this tube, **8** (0.3 mmol, 79 mg) was dissolved in anhydrous THF (2 mL), LiAlH_4_ (0.9 mmol, 34 mg) was then added slowly at 0 °C under N_2_. The reaction was then heated to 100 °C overnight. After cooling to room temperature, the reaction was quenched with a saturated solution of NH_4_Cl. The reaction was then extracted with ether three times. The combined organic extracts were washed with brine, dried with MgSO_4_, filtrated and concentrated in vacuo.^5^ The crude product was purified by flash column chromatography (petroleum ether/ethyl acetate = 20/1) to yield **9** as a white solid (60.5 mg, 81% yield with 94% *ee*). Mp: 68-69 ^o^C. ^1^H NMR (400 MHz, CDCl_3_) δ 7.16 – 7.01 (m, 5H), 6.82 – 6.73 (m, 1H), 6.66 (t, *J* = 7.3 Hz, 1H), 6.56 (d, *J* = 7.8 Hz, 1H), 3.52 (d, *J* = 8.9 Hz, 1H), 3.28 (d, *J* = 9.0 Hz, 1H), 2.98 – 2.82 (m, 2H), 2.79 (s, 3H), 2.12 (dd, *J* = 10.5, 5.0 Hz, 1H), 2.01 – 1.91 (m, 1H), 1.91 – 1.78 (m, 2H). ^13^C NMR (101 MHz, CDCl_3_) δ 152.70, 141.80, 139.27, 137.33, 129.54, 128.64, 127.69, 125.98, 125.88, 123.55, 118.21, 107.25, 71.59, 48.72, 35.88, 35.70, 29.92, 20.21. HRMS (ESI) calculated for C_18_H_20_N: 250.1590 (M+H^+^), found: 250.1588. Enantiomeric excess was determined by HPLC with a Chiralpak ADH column (hexanes/2-propanol = 99.5/0.5, 1.0 mL/min, 254 nm); minor enantiomer *t_R_* = 5.4 min, major enantiomer *t_R_* = 4.9 min. [α]_D_^20^ = 88.1 (*c* = 0.3, CHCl_3_).

6.4 Synthesis of (*R*)-1-methyl-3',4'-dihydro-2'*H*-spiro[indoline-3,1'-naphthalene]-2-thione (**10**).

**8** (79 mg, 0.3 mmol) and Lawesson’s reagent (0.51 equiv) were added into a test tube under N_2_. Then dry toluene (2 mL) was added by syringe. It was sealed and refluxed for 1.5-2 h. After cooling down, the mixture was poured into water. The organic layer was separated and the aqueous layer was extracted with ether. The organic layers were combined, washed with brine, dried over Na_2_SO_4_, concentrated in vacuo and finally purified by flash column chromatography (petroleum ether/ethyl acetate = 20/1) to yield **10** as a white solid (82.3 mg, 98% yield with 93% *ee*).^5^ Mp: 125-127 ^o^C. ^1^H NMR (400 MHz, CDCl_3_) δ 7.35 – 7.26 (m, 1H), 7.18 – 7.03 (m, 5H), 6.97 – 6.88 (m, 1H), 6.34 (d, *J* = 7.8 Hz, 1H), 3.71 (s, 3H), 3.06 (ddd, *J* = 15.9, 10.0, 5.5 Hz, 1H), 2.96 (dt, *J* = 16.7, 5.0 Hz, 1H), 2.51 – 2.37 (m, 1H), 2.37 – 2.23 (m, 1H), 2.20 – 2.02 (m, 1H), 1.84 (ddd, *J* = 13.5, 6.1, 2.9 Hz, 1H). ^13^C NMR (101 MHz, CDCl_3_) δ 211.82, 143.59, 142.10, 137.09, 136.72, 129.23, 128.40, 127.71, 126.83, 126.22, 124.24, 124.13, 109.43, 62.93, 38.13, 31.62, 29.12, 18.87. HRMS (ESI) calculated for C_18_H_18_NS: 280.1154 (M+H^+^), found: 280.1154. Enantiomeric excess was determined by HPLC with a Chiralpak ADH column (hexanes/2-propanol = 98/2, 1.0 mL/min, 254 nm); minor enantiomer *t_R_* = 8.2 min, major enantiomer *t_R_* = 9.0 min. [α]_D_^20^ = -40.4 (*c* = 0.3, CHCl_3_).

6.5 Synthesis of *N*-((3*R*,4'*S*)-1-methyl-2-oxo-4'*H*-spiro[indoline-3,1'-naphthalen]-4'-yl)-*N*-phenylacetamide (**11**).

A flame-dried flask was cooled down to room temperature under argon. To this tube were added **3g** (105.7 mg, 0.3 mmol), DCM (3.0 mL), Et_3_N (83.4 μL, 0.6 mmol) and CH_3_COCl (43.0 μL, 0.6 mmol). The mixture was stirred at room temperature for 18 h. Upon reaction completion (monitored by TLC), the reaction was quenched by saturated NH_4_Cl solution and extracted with DCM. The combined organic layers were Na_2_SO_4_ and concentrated to dryness. The crude product was purified by silica gel column chromatography (petroleum ether/ethyl acetate = 5/1) to afford **11** as a white solid (117.2 mg, 97% yield) with 95% *ee*.^6^ Mp: 216-218 ^o^C. ^1^H NMR (400 MHz, CDCl_3_) δ 7.63 (d, *J* = 7.9 Hz, 1H), 7.33 – 7.21 (m, 5H), 7.11 (t, *J* = 2.8 Hz, 1H), 7.04 (t, *J* = 7.6 Hz, 1H), 6.95 (t, *J* = 7.5 Hz, 1H), 6.86 (dd, *J* = 11.1, 7.0 Hz, 3H), 6.80 (dd, *J* = 7.5, 1.2 Hz, 1H), 6.48 (dd, *J* = 7.8, 1.3 Hz, 1H), 6.11 (dd, *J* = 10.1, 3.6 Hz, 1H), 5.51 (dd, *J* = 10.1, 2.1 Hz, 1H), 3.18 (s, 3H), 1.97 (s, 3H). ^13^C NMR (101 MHz, CDCl_3_) δ 176.68, 171.27, 143.00, 138.71, 135.10, 134.61, 133.85, 129.60, 128.78, 128.70, 128.33, 128.01, 127.43, 127.26, 127.21, 126.73, 124.42, 123.13, 107.95, 53.49, 50.11, 26.55, 23.14. HRMS (EI) calculated for [C_26_H_22_N_2_O_2_]^+^: 394.1681 found: 394.1676. Enantiomeric excess was determined by HPLC with a Chiralpak AD-H column (hexanes/2-propanol = 82/18, 1.0 mL/min, 210 nm); minor enantiomer *t_R_* = 6.9 min, major enantiomer *t_R_* = 8.5 min. [α]_D_^20^ = 59.3 (*c* = 0.3, CHCl_3_).

6.6 Synthesis of (3*R*,4'*S*)-1-methyl-4'-(methyl(phenyl)amino)-4'*H*-spiro[indoline-3,1'-naphthalen]-2-one (**12**).

A flame-dried flask was cooled down to room temperature under argon. The flask equipped with a stir bar was charged with a solution of the **3g** (105.7 mg, 0.3 mmol) in dry THF (3 mL), the mixture was cooled to -40 ^o^C and *^n^*BuLi (0.45 mmol, 1.5 equiv, 2.5 M in hexane) was added dropwise. After one hour, indomethane (38 μL, 0.6 mmol, 2.0 equiv.) was added at -40 ^o^C and stirred for another one hour at this temperature. Upon reaction completion (monitored by TLC), the reaction was quenched by saturated NH_4_Cl solution and extracted with ethyl acetate for three times. The combined organic layers were washed by brine, dried over Na_2_SO_4_ and concentrated to dryness. The residue was purified by column chromatography (petroleum ether/ethyl acetate = 8/1) to afford **12** as a white solid (96.3 mg, 88% yield) with 93% *ee*.^7^ Mp: 145-148 ^o^C. ^1^H NMR (400 MHz, CDCl_3_) δ 7.39 (s, 1H), 7.35 – 7.27 (m, 3H), 7.17 (td, *J* = 7.6, 1.4 Hz, 1H), 7.12 – 6.98 (m, 4H), 6.94 (d, *J* = 7.6 Hz, 2H), 6.78 (t, *J* = 7.2 Hz, 1H), 6.60 (d, *J* = 7.9 Hz, 1H), 6.13 (dd, *J* = 10.0, 3.4 Hz, 1H), 5.89 – 5.78 (m, 1H), 5.71 (dd, *J* = 10.0, 1.9 Hz, 1H), 3.31 (s, 3H), 2.77 (s, 3H). ^13^C NMR (101 MHz, CDCl_3_) δ 177.65, 150.40, 143.52, 135.22, 135.05, 134.94, 129.38, 128.54, 128.16, 127.91, 127.84, 127.74, 127.57, 126.88, 124.50, 123.33, 116.93, 112.88, 108.24, 54.60, 54.12, 32.09, 26.64. HRMS (EI) calculated for [C_25_H_22_N_2_O]^+^: 366.1732 found: 366.1729. Enantiomeric excess was determined by HPLC with a Chiralpak IA column (hexanes/2-propanol = 90/10, 0.7 mL/min, 254 nm); minor enantiomer *t_R_* = 10.1 min, major enantiomer *t_R_* = 11.0 min. [α]_D_^20^ = 7.6 (*c* = 0.3, CHCl_3_).

6.7 Synthesis of (3*R*,4'*S*)-1-methyl-4'-(methyl(phenyl)amino)-4'*H*-spiro[indoline-3,1'-naphthalen]-2-one (**13**).

A flame-dried flask was cooled down to room temperature under argon. The flask equipped with a stir bar was charged with a solution of the **3g** (105.7 mg, 0.3 mmol) in dry THF (3 mL), the mixture was cooled to -40 ^o^C and *^n^*BuLi (0.45 mmol, 1.5 equiv, 2.5 M in hexane) was added dropwise. After one hour, Benzyl bromide (72 μL, 0.6 mmol, 2.0 equiv.) was added at -40 ^o^C and stirred for another 8 hour at this temperature. Upon reaction completion (monitored by TLC), the reaction was quenched by saturated NH_4_Cl solution and extracted with ethyl acetate for three times. The combined organic layers were washed by brine, dried over Na_2_SO_4_ and concentrated to dryness. The residue was purified by column chromatography (petroleum ether/ethyl acetate = 8/1) to afford **13** as a pale yellow solid (83.0 mg, 63% yield with 93% *ee*).^8^ Mp: 207-208 ^o^C. ^1^H NMR (400 MHz, CDCl_3_) δ 7.51 (d, *J* = 7.8 Hz, 1H), 7.33 – 7.16 (m, 7H), 7.15 – 7.06 (m, 2H), 7.02 (qd, *J* = 8.0, 6.6, 2.8 Hz, 4H), 6.98 – 6.90 (m, 2H), 6.76 (t, *J* = 7.2 Hz, 1H), 6.57 (dd, *J* = 7.9, 1.3 Hz, 1H), 6.27 (dd, *J* = 10.0, 3.4 Hz, 1H), 5.98 (d, *J* = 2.5 Hz, 1H), 5.69 (dd, *J* = 10.0, 1.9 Hz, 1H), 4.56 (q, *J* = 17.3 Hz, 2H), 3.29 (s, 3H). ^13^C NMR (101 MHz, CDCl_3_) δ 177.50, 149.39, 143.58, 139.99, 135.25, 135.07, 135.03, 129.21, 128.58, 128.54, 128.01, 127.96, 127.67, 127.62, 126.90, 126.76, 126.01, 124.53, 123.33, 117.66, 114.77, 108.24, 55.71, 54.16, 49.47, 26.72. HRMS (ESI) calculated for C_31_H_27_N_2_O: 443.2118 (M+H^+^), found: 443.2124. Enantiomeric excess was determined by HPLC with a Chiralpak IA column (hexanes/2-propanol = 90/10, 1.0 mL/min, 254 nm); minor enantiomer *t_R_* = 8.0 min, major enantiomer *t_R_* = 10.3 min. [α]_D_^20^ = -0.6 (*c* = 0.3, CHCl_3_).

7. **Non-linear effect experiments**

Non-linear effect reactions were set up with substrate **3a** (0.2 mmol), catalyst and **M4** with varied *ee* following the standard procedure as described in previous section. In this case, the amount of palladium precatalyst and **M4** enantiomers was weighed on an analytical balance, then add DCM (0.5 mL). The solution was stirred for 40 min at room temperature. Then other reactants and DCM (0.5 ml) were added to the solution. The reactions were allowed to run for 15 h at 80 ^o^C, purification as described in standard procedure. Each experiment was repeated three times. The relationship between enantiomeric excess of **M4** ligand and product is listed here.

**Table S1.** Non-linear effect experiments

| **Ligand**  ***ee*** | **(*R,R*)-M4 amount**  **weighed (mg)** | **(*S*,*S*)-M4 amount**  **weighed (mg)** | **Product first *ee*** | **Product**  **second *ee*** | **Product**  **third *ee*** | **Product**  **average *ee*** |
| --- | --- | --- | --- | --- | --- | --- |
| 0 | 5.8 | 5.8 | -2.7% | 0.9% | -2.3% | -1.4% |
| 20% | 6.9 | 4.6 | 19.9% | 18.4% | 17.6% | 18.6% |
| 40% | 8.2 | 3.5 | 43.9% | 39% | 40.4% | 41.1% |
| 60% | 9.2 | 2.3 | 66.8% | 54.8% | 56.6% | 59.4% |
| 80% | 10.8 | 1.2 | 85.2% | 81.2% | 82.3% | 82.9% |
| 100% | 11.7 | 0 | 97.4% | 95.3% | 96.8% | 96.5% |

| M3, ee% | 3e, ee% |
| --- | --- |
| 100 | 96.5 |
| 80 | 82.9 |
| 60 | 59.4 |
| 40 | 41.1 |
| 20 | 18.6333 |
| 0 | -1.36667 |

8. **Kinetic Studies**

**A: Order in Catalyst**

The order in [*η*-PhC_3_H_4_PdCl]_2_/**M4** was determined according to the following procedure: In an oven-dried 10 mL Schlenk tube equipped with a stir bar, [*η*-PhC_3_H_4_PdCl]_2_ (from 0.005 mmol to 0.015 mmol), **M4** (from 0.014 mmol to 0.042 mmol) and DCM (0.5 mL) was added to the tube under nitrogen atmosphere, and stirred at room temperature for 40 min. Then **1a** (77.4 mg, 0.2 mmol), **2a** (48.5 mg, 0.4 mmol) and Ag_3_PO_4_ (83.6 mg, 0.2 mmol, 1.0 equiv.), Na_2_HPO_4_·12H_2_O (143.2 mg, 0.3 mmol, 2.0 equiv) and DCM (0. 5 ml) were added to the solution, and stirred at 80 °C. Subsequently, appropriate time to take sample 1-2 drops to check yield by GC.

**B: Kinetic Order in 1a**

The order in **1a** was determined according to the following procedure: In an oven-dried 10 mL Schlenk tube equipped with a stir bar, [*η*-PhC_3_H_4_PdCl]_2_ (2.5 mg, 2.5 mol%), **M4** (11.5 mg, 7 mol%) and DCM (0.5 mL) was added to the tube under nitrogen atmosphere, and stirred at room temperature for 40 min. Then **1a** (0.12 mmol to 0.2 mmol), **2a** (48.5 mg, 0.4 mmol) and Ag_3_PO_4_ (83.6 mg, 0.2 mmol, 1.0 equiv.), Na_2_HPO_4_·12H_2_O (143.2 mg, 0.3 mmol, 2.0 equiv) and DCM (0. 5 ml) were added to the solution, and stirred at 80 °C. Subsequently, appropriate time to take sample 1-2 drops to check yield by GC.

**A: Kinetic Order in 2a**

The order in **2a** was determined according to the following procedure: In an oven-dried 10 mL Schlenk tube equipped with a stir bar, [*η*-PhC_3_H_4_PdCl]_2_ (2.5 mg, 2.5 mol%), **M4** (11.5 mg, 7 mol%) and DCM (0.5 mL) was added to the tube under nitrogen atmosphere, and stirred at room temperature for 40 min. Then **1a** (77.4 mg, 0.2 mmol), **2a** (48.5 mg to 72.7 mg, 0.2 mmol to 0.6 mmol) and Ag_3_PO_4_ (83.6 mg, 0.2 mmol, 1.0 equiv), Na_2_HPO_4_·12H_2_O (143.2 mg, 0.3 mmol, 2.0 equiv) and DCM (0. 5 ml) were added to the solution, and stirred at 80 °C. Subsequently, appropriate time to take sample 1-2 drops to check yield by GC.

9. **X-Ray Structure and Crystal Data of 3g**

| **Table 1 Crystal data and structure refinement for 3g.** | |
| --- | --- |
| Identification code | exp_4901_auto |
| Empirical formula | C_24_H_20_N_2_O |
| Formula weight | 352.42 |
| Temperature/K | 172(1) |
| Crystal system | monoclinic |
| Space group | P2_1_ |
| a/Å | 6.3172(5) |
| b/Å | 12.6957(9) |
| c/Å | 11.7308(6) |
| α/° | 90 |
| β/° | 96.772(6) |
| γ/° | 90 |
| Volume/Å^3^ | 934.26(11) |
| Z | 2 |
| ρ_calc_g/cm^3^ | 1.253 |
| μ/mm^‑1^ | 0.604 |
| F(000) | 372.0 |
| Crystal size/mm^3^ | 0.13 × 0.11 × 0.08 |
| Radiation | Cu Kα (λ = 1.54184) |
| 2Θ range for data collection/° | 7.59 to 133.988 |
| Index ranges | -7 ≤ h ≤ 7, -15 ≤ k ≤ 15, -14 ≤ l ≤ 14 |
| Reflections collected | 19445 |
| Independent reflections | 3325 [R_int_ = 0.1507, R_sigma_ = 0.1092] |
| Data/restraints/parameters | 3325/1/245 |
| Goodness-of-fit on F^2^ | 1.041 |
| Final R indexes [I>=2σ (I)] | R_1_ = 0.0683, wR_2_ = 0.1713 |
| Final R indexes [all data] | R_1_ = 0.0885, wR_2_ = 0.1939 |
| Largest diff. peak/hole / e Å^-3^ | 0.26/-0.26 |
| Flack parameter | -0.3(4) |

**Table 2 Fractional Atomic Coordinates (×10^4^) and Equivalent Isotropic Displacement Parameters (Å^2^×10^3^) for 3g. U_eq_ is defined as 1/3 of the trace of the orthogonalised U_IJ_ tensor.**

|  | | | | |
| --- | --- | --- | --- | --- |
| **Atom** | ***x*** | ***y*** | ***z*** | **U(eq)** |
| O1 | 7500(7) | 4322(3) | 1296(3) | 54.4(10) |
| N1 | 5803(7) | 2746(3) | 873(3) | 45.1(11) |
| N2 | 7512(8) | 6149(3) | 3086(4) | 50.3(11) |
| C1 | 3386(9) | 2691(4) | 2171(4) | 48.0(13) |
| C2 | 1705(10) | 2280(5) | 2688(4) | 52.3(14) |
| C3 | 810(11) | 1335(5) | 2270(5) | 59.9(16) |
| C4 | 1585(10) | 814(5) | 1364(5) | 57.4(15) |
| C5 | 3267(10) | 1224(4) | 840(4) | 52.9(14) |
| C6 | 4132(9) | 2162(4) | 1259(4) | 46.0(13) |
| C7 | 6996(10) | 2435(5) | -76(4) | 57.1(15) |
| C8 | 6173(9) | 3651(4) | 1467(4) | 44.8(12) |
| C9 | 4653(8) | 3688(4) | 2424(4) | 44.3(12) |
| C10 | 3278(10) | 4662(4) | 2290(4) | 48.9(13) |
| C11 | 3765(10) | 5546(4) | 2852(4) | 50.5(13) |
| C12 | 5797(9) | 5661(4) | 3669(4) | 47.7(13) |
| C13 | 6583(9) | 4618(4) | 4140(4) | 48.7(13) |
| C14 | 7950(10) | 4590(5) | 5183(4) | 56.5(15) |
| C15 | 8774(11) | 3662(6) | 5618(4) | 63.0(16) |
| C16 | 8271(10) | 2721(5) | 5059(5) | 57.4(15) |
| C17 | 6890(9) | 2729(5) | 4037(4) | 49.4(14) |
| C18 | 6067(8) | 3669(4) | 3583(4) | 45.1(12) |
| C19 | 7257(9) | 7153(4) | 2626(4) | 45.5(13) |
| C20 | 8349(9) | 7415(4) | 1693(4) | 49.9(13) |
| C21 | 8168(10) | 8412(4) | 1218(5) | 53.9(14) |
| C22 | 6916(10) | 9176(5) | 1655(5) | 56.8(15) |
| C23 | 5836(11) | 8917(4) | 2584(5) | 56.7(15) |
| C24 | 6001(10) | 7930(5) | 3064(5) | 56.4(15) |

**Table 3 Anisotropic Displacement Parameters (Å2×103) for 3g. The Anisotropic displacement factor exponent takes the form: -2π2[h2a*2U11+2hka*b*U12+…].**

| **Atom** | **U_11_** | **U_22_** | **U_33_** | **U_23_** | **U_13_** | **U_12_** |
| --- | --- | --- | --- | --- | --- | --- |
| O1 | 63(3) | 62(2) | 39.3(18) | 1.8(17) | 10.5(16) | -6(2) |
| N1 | 49(3) | 58(3) | 29.0(18) | -0.5(18) | 9.4(17) | 0(2) |
| N2 | 50(3) | 54(3) | 47(2) | 0(2) | 7.6(19) | 3(2) |
| C1 | 54(3) | 57(3) | 32(2) | 3(2) | 4(2) | 2(3) |
| C2 | 59(4) | 63(4) | 36(2) | 8(3) | 9(2) | -1(3) |
| C3 | 59(4) | 70(4) | 49(3) | 16(3) | 1(3) | -6(3) |
| C4 | 67(4) | 54(3) | 48(3) | 5(3) | -2(3) | -2(3) |
| C5 | 63(4) | 55(3) | 39(3) | 2(2) | 0(2) | 3(3) |
| C6 | 52(3) | 49(3) | 36(2) | 3(2) | 3(2) | 0(2) |
| C7 | 64(4) | 73(4) | 36(3) | -2(3) | 13(2) | 7(3) |
| C8 | 55(3) | 48(3) | 31(2) | 4(2) | 3(2) | -2(3) |
| C9 | 52(3) | 51(3) | 30(2) | 3(2) | 7(2) | 4(3) |
| C10 | 52(3) | 59(3) | 34(2) | 2(2) | -1(2) | -2(3) |
| C11 | 54(3) | 53(3) | 44(3) | 6(2) | 6(2) | 5(3) |
| C12 | 53(3) | 51(3) | 39(3) | -3(2) | 9(2) | -8(3) |
| C13 | 56(3) | 58(3) | 33(2) | 2(2) | 8(2) | -1(3) |
| C14 | 62(4) | 70(4) | 35(3) | 0(3) | -5(2) | -1(3) |
| C15 | 67(4) | 86(4) | 34(3) | 7(3) | -3(2) | 1(4) |
| C16 | 63(4) | 69(4) | 40(3) | 17(3) | 7(2) | 7(3) |
| C17 | 55(4) | 58(3) | 36(2) | 3(2) | 8(2) | 5(3) |
| C18 | 52(3) | 56(3) | 26(2) | 0(2) | 4(2) | -2(3) |
| C19 | 53(3) | 48(3) | 35(2) | -5(2) | 3(2) | -2(2) |
| C20 | 55(4) | 54(3) | 41(3) | -4(2) | 7(2) | -1(3) |
| C21 | 53(3) | 61(4) | 46(3) | 1(2) | 1(2) | -1(3) |
| C22 | 58(4) | 56(3) | 55(3) | 6(3) | 0(3) | -2(3) |
| C23 | 59(4) | 58(4) | 54(3) | -4(3) | 8(3) | 5(3) |
| C24 | 61(4) | 60(3) | 48(3) | -10(3) | 6(3) | 0(3) |

| **Table 4 Bond Lengths for 3g.** | | | | | | |
| --- | --- | --- | --- | --- | --- | --- |
| **Atom** | **Atom** | **Length/Å** | | **Atom** | **Atom** | **Length/Å** |
| O1 | C8 | 1.228(6) |  | C10 | C11 | 1.320(8) |
| N1 | C6 | 1.408(7) |  | C11 | C12 | 1.515(8) |
| N1 | C7 | 1.471(7) |  | C12 | C13 | 1.496(8) |
| N1 | C8 | 1.350(6) |  | C13 | C14 | 1.412(7) |
| N2 | C12 | 1.484(7) |  | C13 | C18 | 1.391(7) |
| N2 | C19 | 1.386(7) |  | C14 | C15 | 1.362(9) |
| C1 | C2 | 1.386(8) |  | C15 | C16 | 1.381(9) |
| C1 | C6 | 1.392(7) |  | C16 | C17 | 1.397(8) |
| C1 | C9 | 1.508(8) |  | C17 | C18 | 1.383(8) |
| C2 | C3 | 1.390(9) |  | C19 | C20 | 1.401(8) |
| C3 | C4 | 1.390(9) |  | C19 | C24 | 1.401(8) |
| C4 | C5 | 1.389(9) |  | C20 | C21 | 1.382(8) |
| C5 | C6 | 1.377(7) |  | C21 | C22 | 1.388(9) |
| C8 | C9 | 1.562(7) |  | C22 | C23 | 1.392(9) |
| C9 | C10 | 1.509(8) |  | C23 | C24 | 1.373(8) |
| C9 | C18 | 1.537(6) |  |  |  |  |

**Table 5 Bond Angles for** **3g.**

| **Atom** | **Atom** | **Atom** | **Angle/˚** |  | **Atom** | **Atom** | **Atom** | **Angle/˚** |
| --- | --- | --- | --- | --- | --- | --- | --- | --- |
| C6 | N1 | C7 | 124.9(4) |  | C11 | C10 | C9 | 123.2(5) |
| C8 | N1 | C6 | 111.9(4) |  | C10 | C11 | C12 | 121.8(5) |
| C8 | N1 | C7 | 123.1(5) |  | N2 | C12 | C11 | 111.0(4) |
| C19 | N2 | C12 | 120.4(4) |  | N2 | C12 | C13 | 108.2(5) |
| C2 | C1 | C6 | 120.1(5) |  | C13 | C12 | C11 | 111.6(4) |
| C2 | C1 | C9 | 130.3(5) |  | C14 | C13 | C12 | 119.0(5) |
| C6 | C1 | C9 | 109.7(5) |  | C18 | C13 | C12 | 122.9(4) |
| C1 | C2 | C3 | 118.1(5) |  | C18 | C13 | C14 | 118.0(5) |
| C4 | C3 | C2 | 121.0(6) |  | C15 | C14 | C13 | 120.9(5) |
| C5 | C4 | C3 | 121.1(5) |  | C14 | C15 | C16 | 120.9(5) |
| C6 | C5 | C4 | 117.3(5) |  | C15 | C16 | C17 | 119.1(5) |
| C1 | C6 | N1 | 109.1(4) |  | C18 | C17 | C16 | 120.4(5) |
| C5 | C6 | N1 | 128.5(5) |  | C13 | C18 | C9 | 118.8(5) |
| C5 | C6 | C1 | 122.4(5) |  | C17 | C18 | C9 | 120.5(5) |
| O1 | C8 | N1 | 125.9(5) |  | C17 | C18 | C13 | 120.7(4) |
| O1 | C8 | C9 | 125.9(5) |  | N2 | C19 | C20 | 118.5(5) |
| N1 | C8 | C9 | 108.2(4) |  | N2 | C19 | C24 | 123.3(5) |
| C1 | C9 | C8 | 101.0(4) |  | C20 | C19 | C24 | 118.2(5) |
| C1 | C9 | C10 | 112.4(4) |  | C21 | C20 | C19 | 120.5(5) |
| C1 | C9 | C18 | 113.6(4) |  | C20 | C21 | C22 | 121.1(6) |
| C10 | C9 | C8 | 110.2(4) |  | C21 | C22 | C23 | 118.5(6) |
| C10 | C9 | C18 | 111.8(4) |  | C24 | C23 | C22 | 121.1(6) |
| C18 | C9 | C8 | 107.1(4) |  | C23 | C24 | C19 | 120.7(6) |

**Table 6 Torsion Angles for 3g.**

| **A** | **B** | **C** | **D** | **Angle/˚** |  | **A** | **B** | **C** | **D** | **Angle/˚** |
| --- | --- | --- | --- | --- | --- | --- | --- | --- | --- | --- |
| O1 | C8 | C9 | C1 | 178.0(5) |  | C8 | C9 | C10 | C11 | -95.0(6) |
| O1 | C8 | C9 | C10 | 59.0(6) |  | C8 | C9 | C18 | C13 | 96.3(5) |
| O1 | C8 | C9 | C18 | -62.8(6) |  | C8 | C9 | C18 | C17 | -80.7(6) |
| N1 | C8 | C9 | C1 | -3.1(5) |  | C9 | C1 | C2 | C3 | 179.6(5) |
| N1 | C8 | C9 | C10 | -122.1(4) |  | C9 | C1 | C6 | N1 | -0.7(6) |
| N1 | C8 | C9 | C18 | 116.1(5) |  | C9 | C1 | C6 | C5 | -179.9(4) |
| N2 | C12 | C13 | C14 | 79.9(6) |  | C9 | C10 | C11 | C12 | 1.4(8) |
| N2 | C12 | C13 | C18 | -98.7(6) |  | C10 | C9 | C18 | C13 | -24.5(7) |
| N2 | C19 | C20 | C21 | -179.0(5) |  | C10 | C9 | C18 | C17 | 158.5(5) |
| N2 | C19 | C24 | C23 | 178.9(5) |  | C10 | C11 | C12 | N2 | 95.5(6) |
| C1 | C2 | C3 | C4 | 0.3(8) |  | C10 | C11 | C12 | C13 | -25.3(7) |
| C1 | C9 | C10 | C11 | 153.2(5) |  | C11 | C12 | C13 | C14 | -157.7(5) |
| C1 | C9 | C18 | C13 | -153.1(5) |  | C11 | C12 | C13 | C18 | 23.8(7) |
| C1 | C9 | C18 | C17 | 29.9(7) |  | C12 | N2 | C19 | C20 | -152.4(5) |
| C2 | C1 | C6 | N1 | 178.9(4) |  | C12 | N2 | C19 | C24 | 29.2(7) |
| C2 | C1 | C6 | C5 | -0.4(8) |  | C12 | C13 | C14 | C15 | -177.3(5) |
| C2 | C1 | C9 | C8 | -177.3(5) |  | C12 | C13 | C18 | C9 | 0.9(8) |
| C2 | C1 | C9 | C10 | -59.9(7) |  | C12 | C13 | C18 | C17 | 177.9(5) |
| C2 | C1 | C9 | C18 | 68.5(7) |  | C13 | C14 | C15 | C16 | -0.9(9) |
| C2 | C3 | C4 | C5 | -0.5(8) |  | C14 | C13 | C18 | C9 | -177.6(5) |
| C3 | C4 | C5 | C6 | 0.2(8) |  | C14 | C13 | C18 | C17 | -0.6(8) |
| C4 | C5 | C6 | N1 | -178.9(5) |  | C14 | C15 | C16 | C17 | -0.3(9) |
| C4 | C5 | C6 | C1 | 0.2(7) |  | C15 | C16 | C17 | C18 | 1.0(9) |
| C6 | N1 | C8 | O1 | -178.2(5) |  | C16 | C17 | C18 | C9 | 176.4(5) |
| C6 | N1 | C8 | C9 | 2.9(5) |  | C16 | C17 | C18 | C13 | -0.5(8) |
| C6 | C1 | C2 | C3 | 0.1(7) |  | C18 | C9 | C10 | C11 | 23.9(8) |
| C6 | C1 | C9 | C8 | 2.2(5) |  | C18 | C13 | C14 | C15 | 1.3(9) |
| C6 | C1 | C9 | C10 | 119.6(5) |  | C19 | N2 | C12 | C11 | 61.0(6) |
| C6 | C1 | C9 | C18 | -112.1(5) |  | C19 | N2 | C12 | C13 | -176.3(4) |
| C7 | N1 | C6 | C1 | 179.8(5) |  | C19 | C20 | C21 | C22 | 0.4(8) |
| C7 | N1 | C6 | C5 | -1.0(8) |  | C20 | C19 | C24 | C23 | 0.5(8) |
| C7 | N1 | C8 | O1 | 0.6(8) |  | C20 | C21 | C22 | C23 | -0.2(8) |
| C7 | N1 | C8 | C9 | -178.3(4) |  | C21 | C22 | C23 | C24 | 0.1(9) |
| C8 | N1 | C6 | C1 | -1.5(6) |  | C22 | C23 | C24 | C19 | -0.3(9) |
| C8 | N1 | C6 | C5 | 177.7(5) |  | C24 | C19 | C20 | C21 | -0.5(8) |

**Table 7 Hydrogen Atom Coordinates (Å×10^4^) and Isotropic Displacement Parameters (Å^2^×10^3^) for 3g.**

| **Atom** | ***x*** | ***y*** | ***z*** | **U(eq)** |
| --- | --- | --- | --- | --- |
| H2 | 8698.87 | 5799.33 | 3031.23 | 60 |
| H2A | 1178.54 | 2633.94 | 3310.17 | 63 |
| H3 | -349.02 | 1041.46 | 2610.01 | 72 |
| H4 | 955.14 | 166.73 | 1097.47 | 69 |
| H5 | 3797.18 | 871.63 | 217.8 | 64 |
| H7A | 7499.65 | 3066.25 | -441.97 | 86 |
| H7B | 6062.67 | 2029.62 | -642.48 | 86 |
| H7C | 8219.82 | 2000.47 | 222.1 | 86 |
| H10 | 1999.42 | 4637.67 | 1775.75 | 59 |
| H11 | 2814.9 | 6127.17 | 2742.13 | 61 |
| H12 | 5502.39 | 6122.88 | 4321.87 | 57 |
| H14 | 8300.88 | 5226.6 | 5586.42 | 68 |
| H15 | 9706.65 | 3661.87 | 6314.89 | 76 |
| H16 | 8855.76 | 2076.99 | 5365.78 | 69 |
| H17 | 6515.04 | 2085.84 | 3651.73 | 59 |
| H20 | 9220.58 | 6903.57 | 1384.4 | 60 |
| H21 | 8911.63 | 8576.14 | 583.1 | 65 |
| H22 | 6799.03 | 9861.32 | 1327.4 | 68 |
| H23 | 4970.71 | 9431.52 | 2891.64 | 68 |
| H24 | 5254.21 | 7771.16 | 3700.04 | 68 |

**10. References**

(1) Zhou, B.; Wang, H.; Cao, Z.-Y.; Zhu, J.-W.; Liang, R.-X.; Hong, X.; Jia, Y.-X. Dearomative 1,4-difunctionalization of naphthalenes via palladium-catalyzed tandem Heck/Suzuki coupling reaction, *Nat. Commun.*, **2020**, *11*, 4380.
(2) Zhang, Z.; Chen, P.; Li, W.; Niu, Y.; Zhao, X.; Zhang, J. A New Type of Chiral

Sulfinamide Monophosphine Ligands: Stereodivergent Synthesis and Application

in Enantioselective Gold(I)-Catalyzed Cycloaddition Reactions. *Angew. Chem. Int. Ed.*, **2014**, *53*, 4350-4354.

(3) Sato, T.; Watanabe, S.; Kiuchi, H.; Oi, S.; Inoue, Y. Hydrogenation of olefins using water and zinc metal catalyzed by a rhodium complex. *Tetrahedron Lett.*, **2006**, *47*, 7703-7705.
(4) Yang, P.; Zheng, C.; Nie, Y.-H.; You, S.-L. Palladium-catalyzed dearomative 1,4

difunctionalization of naphthalenes. *Chem. Sci.*, **2020**, *11*, 6830.

(5) Wang, C.; Liu, L. *Org. Chem. Front.*, **2021**, *8*, 1454-1460.
(6) Chaturvedi, J.; Haldar, C.; Bisht, R.; Pandey, G.; Chattopadhyay, B. Meta Selective C–H Borylation of Sterically Biased and Unbiased Substrates Directed by Electrostatic Interaction. *J. Am. Chem. Soc.*, **2021**, *143*, 7604-7611.
(7) Semeniuk, T.; Dudas, T.; Okeh, E.; Felesky, T.; Hamel, J.-D. Photocatalytic Defluorinative α-Aminoalkylation of Allylic Difluorides. *J. Org. Chem.*, **2024**, *89*, 13669-13677.
(8) Schmid, S.; Röttgen, M.; Thewalt, U.; Austel, V. Synthesis and conformational properties of 2,6-bis-anilino-3-nitropyridines. *Org. Biomol. Chem.*, **2005**, *3*, 3408-3421.
11. **^1^H , ^19^F, ^31^P, ^13^C NMR**
